# Epigenetic alterations impede epithelial-mesenchymal transition by modulating centrosome amplification and Myc/RAS axis in triple negative breast cancer cells

**DOI:** 10.1038/s41598-023-29712-8

**Published:** 2023-02-11

**Authors:** Laxmidhar Das

**Affiliations:** Department of Biotechnology and Bioengineering, Institute of Advanced Research (IAR), The University for Innovation, Koba Institutional Area, Gandhinagar, Gujarat 382426 India

**Keywords:** Biochemistry, Cancer, Cell biology, Molecular biology, Oncology

## Abstract

Alterations in centrosome proteins may result in centrosome abnormalities such as disorganized spindles and centrosome amplification, leading to aneuploidy and genomic instability. Centrosomes exhibit unique epigenetic properties in which structural or positional information is propagated through somatic lineage by non-genetic pathways. Excessive centrosome amplification in breast cancer is accompanied by efficient clustering and loss of E-cadherin, indicating an important adaptive mechanism of cancer. This study sought to elucidate the effect of epigenetic alterations on centrosome amplification, epithelial-mesenchymal transition (EMT) and apoptosis in triple negative human breast adenocarcinoma derived MDA-MB-231 cell line. The results obtained here show that siRNA mediated silencing of DNMT1 and specific inhibition of HDAC1 & HDAC2 by Tricostatin A (TSA) synergistically inhibit cell proliferation through modulation of centrosome proteins γ-tubulin, TUBGCP2 and pericentrin. In addition, induction of apoptosis was observed by downregulation of Bcl2, upregulation of Bax and activation of PARP cleavage. Inhibition of EMT was confirmed through upregulation of E-cadherin and downregulation of N-cadherin and vimentin. Similarly, downregulation of Myc, RAS and CDK2, which plays important roles in proliferation and survival, was observed. Nuclear protein analysis revealed downregulation in the nuclear translocation of E2F1, which regulates centrosome amplification and metastasis in breast cancer. In conclusion, this study confirmed the role of epigenetic regulators in centrosome amplification and suggests that inhibition of DNA methylation and histone deacetylation-mediated chromatin remodelling synergistically disrupt EMT through modulation of centrosome amplification and Myc/RAS axis to potentiate apoptosis and attenuate cell proliferation in triple negative breast cancer cells.

## Introduction

Microtubule nucleation to form mitotic spindle and to regulate even division of chromosomes is performed by centrosomes during cell division^1^. Centrosomes represent exclusive epigenetic properties in which structural or positional information is propagated through somatic lineage by nongenetic pathways^2^. Several studies have identified number of important centrosome proteins by mass spectrometry analysis, including structural proteins (PCM1, α-tubulin, β-tubulin, γ-tubulin, centrin, pericentrin, ninein, etc.) and regulatory molecules (Cdc2, Cdk1, Plk1, Nek2, etc.)^3^. Although, α-tubulin and β-tubulin are not centrosome markers, but found in the centrosome, because centrosome organizes microtubules made up of α-tubulin and β-tubulin. Further, centrosome is composed of pericentriolar material (PCM) on the outside and γ-tubulin is concentrated in the PCM of interphase cells. γ-tubulin is a centrosome protein and an important key component for microtubule nucleation and stabilization. γ-tubulin forms complexes with TUBGCP2 and TUBGCP3 to form γ-tubulin small complex (γTuSC). γTuSC form complexes with other cytoplasmic complex proteins, including TUBGCPs 4, 5, and 6 form γ-tubulin ring complex (γTuRC)^4^. Centrosome abnormalities are recurring features of human tumors because in vivo centrosome amplification enhances tumor initiation and progression^5^. Centrosome amplification in cancer was reported over a century ago^6^. Centrosome amplification in human tumorigenesis can occur prior to transformation and increases upon p53 loss in tumorigenesis, which further support that the centrosome amplification in humans can contribute to tumor initiation and progression^7^. Centrosome amplification is not observed in normal human cells, including cells of the breast tissue, in contrast to their malignant counterparts^8^. Centrosome amplification is poorly tolerated by nontransformed cells and extra centrosomes are spontaneously lost in normal cells. Increased centrosome amplification in highly aggressive cancers supports the possibility that additional centrosomes may benefit from advantageous features that promote tumor progression^9^. Cell lines with high centrosome amplification express high levels of centrosome structural proteins (centrin-2 and γ-tubulin) and proteins known to cause dysregulation of centrosome amplification^10^. Many studies have reported γ-tubulin is used to localize and quantify centrosomes and overexpression of γ-tubulin protein can lead to centrosome aberration, as well as increased expression of γ-tubulin has been reported in preinvasive lesions and breast carcinomas^11^. Increased expression of α-tubulin and γ-tubulin has been reported in atypical ductal hyperplasia and breast carcinoma^12^. Likewise pericentrin is another centrosome protein found in association with PCM along with γTuRC^13^. Pericentrin and γ-tubulin assemble into a unique centrosome lattice representing higher order organization of microtubule nucleation sites in centrosomes^14^. Overexpression of PCM components such as pericentrin and γ-tubulin leads to centrosome amplification^15^.

Furthermore, centrosome amplification is closely associated with epithelial to mesenchymal transition (EMT) in aggressive TNBC^3,16,17^. E-cadherin; a member of the orchestra of EMT is required for centrosome and spindle orientation. Higher centrosome amplification in breast cancer is accompanied by effective clustering and loss of E-cardherrin, representing an important adaptive mechanism for centrosome amplification^18,19^. Vimentin is another important member participate in EMT, contributes by mediating cytoskeletal organization and remains associated with centrosome via intermediate filaments to aid centrosome amplification^20,21^. In addition, centrosome amplification is reported to be induced by deregulation of E2F^22^. E2Fs are family of transcription factors involved in cell cycle regulation and altered expression of E2Fs in breast cancer influences survival outcome, as patients with higher expression of E2F1 and cyclin A exhibit very less disease-free survival^23^. However, the accumulation of E2F and active cyclin E/Cdk2 is induced by cooperation of Myc and Ras^24^. Myc mediates EMT in TNBC cells, as well as KRas signals centrosome amplification and cell proliferation in mammary epithelial cells^25,26^. In this study, it was speculated that, epigenetic alteration may be able to modulate EMT through modulation of important centrosome proteins. Thus, targeting centrosome amplification and EMT by epigenetic modulators may lead to activation of apoptosis and attenuation of cell proliferation in TNBC. Therefore, this study aimed to elucidate the effect of epigenetic changes on centrosome amplification, EMT and Myc/Ras mediated cell proliferation in TNBC.

## Materials and methods

### Chemicals

All the chemicals used in this study were molecular biology grade as well as endotoxin free and used without further purification. Culture media, fetal bovine serum (FBS) and trypsin were purchased from Himedia and antibiotic from GIBCO. Trichostatin A (TSA), reagents for RNA isolation, cDNA synthesis Kit, Syber green for qRT-PCR, gene specific primer pairs were obtained from Sigma-Aldrich, St. Louis, MO, USA. Anti-DNMT1, anti-HDAC1, mouse monoclonal anti-β-actin and Goat anti-rabbit IgG-HRP antibodies, DNMT1 siRNA and scrambled (control) siRNA were purchased from Santa-Cruz Biotechnology. Anti-PARP (cleaved), anti-HDAC2, anti-E-cadherin, anti-N-cadherin, anti-vimentin, anti-α-tubulin, anti-γ-tubulin, anti-pericentrin, anti-TUBGCP2, anti-c-Myc, anti-Ras, anti-Cdk2 and anti-E2F1, anti-Bcl2 and anti-Bax antibodies were obtained from abcam.

### Cell culture, siRNA transfection and drug treatment

Triple negative human breast adenocarcinoma derived MDA-MB-231 cell line was purchased from National Centre for Cell Science (NCCS), Pune, India. Cells were cultured in Leibovitz’s medium (L-15) supplemented with 10% (v/v) Fetal bovine serum (FBS), penicillin (100 unit/ml) and streptomycin (0.1 mg/ml) in the humidified atmosphere of 5% CO_2_ at 37 °C. Breast cancer cell lines derived from TNBC patients (like MDA-MB-468, MDA-MB-231) exhibit (a) higher incidence and severity of centrosome amplification (b) higher expression of centrosome proteins (pericentrin, centrin-2, γ-tubulin) and centrosome amplification markers (Plk4 and cyclin E), compared to non-TNBC-derived cell lines (like MCF-7)^27^. MDA-MB-231 cells having mutant p53 in both alleles demonstrate amplified centrosomes, exhibit an advanced breast cancer phenotype with aggressive metastatic properties, loss of p53-mediated checkpoint control and a high degree of genomic instability^28,29^. The cell lines CAL-51 and MDA-MB-231 have been reported to serve as low and high chromosome instability (CIN) models respectively. However, MDA-MB-231 cells show a significantly higher degree of centrosome amplification^30^. MDA-MB-231 cell line show invasive in vitro, but when introduced directly into the circulation, the cell line has proven useful in experimental metastasis models. Cell lines expressing the HER2 subtype, including SKBR3 and MDA-MB-453 cells, have low tumorigenic potential. The MDA-MB-435 cell line is considered a questionable use of a melanoma cell line as a model for human breast cancer^31^. MDA-MB-231 cells having amplified centrosomes show increased migration ability^27^. The MDA-MB-231 cells possesses lower expression of epithelial marker E-cadherin and higher expression of mesenchymal marker vimentin, compared to the MCF-7 and MDA-MB-468 cells, thus exhibit representative EMT associated with metastasis^32^. Additionally, HDAC inhibitors have been reported to selectively target MDA-MB-231 TNBC cell proliferation and survival in vitro and tumorigenesis in vivo compared to other TNBC and non-TNBC cells^33^. Therefore, MDA-MB-231 cell line was used in this study.

Histone modification and chromatin remodeling enzymes are recruited by transcriptional activators and repressors to remodel chromatin, ultimately altering the transcriptional activity of multiple genes. This study focused on chromatin remodeling mediated by DNA methylation and histone deacetylation, accordingly epigenetic alterations were achieved by siRNA-mediated silencing of DNMT1 and specific inhibition of HDAC1 & HDAC2 by TSA. Transfection of DNMT1 siRNA was performed with Lipofectamine 2000 Transfection reagent (Invitrogen) and optiMEM Transfection media (Invitrogen) to knockdown DNMT1 expression according to the manufacturer's protocol. As previously reported, 300 nM of DNMT1 siRNA was taken as the optimal siRNA concentration to efficiently inhibit expression at the protein level^34–36^. To determine whether there is a synergistic effect due to inhibition of HDAC1 & HDAC2 expression by TSA and siRNA-mediated knockdown of DNMT1, cells were transfected with DNMT1 siRNA and then treated with TSA at an appropriate concentration after 6 h of siRNA transfection. As previously reported, for the MDA-MB-231 cell line an optimal concentration of TSA sufficient to significantly reduce HDAC1 and HDAC2 expression after treatment was used^36,37^. As per the requirement of experiment, cells were either cultured in plates or in six well tissue culture dishes. Accordingly, one well was treated with TSA (150 µM), one well was transfected with DNMT1 siRNA, one well was transfected with DNMT1 siRNA and treated with TSA, as well as one well was transfected with scrambled (control) siRNA. Besides, one well without any treatment was taken as control. Cells were harvested and all the experiments were done after 24 h of TSA treatment.

### In-silico analysis of the expression of centrosome proteins γ-tubulin and pericentrin in breast cancer

Pre-experimental in-silico analysis of the expression profiles of γ-tubulin and pericentrin in breast cancer from up-to-date publicly available gene expression data was evaluated using online bioinformatics tools such as Kaplan–Meier plotter. Overall survival curves for patients with altered expression of γ-tubulin and pericentrin were calculated from Kaplan–Meier plots using the online KMPLOT tool for individual patients.

### MTT assay

The detection of normal metabolic activity within the cells of a cell population reflects the state of cell proliferation. Therefore, to determine the individual or synergistic effects of DNMT1 silencing and specific inhibition of HDAC1 and HDAC2 on cell proliferation, cells were transfected with DNMT1 siRNA followed by TSA treatment. MTT assay was performed as described previously to estimate the viable cell count after 24 h of TSA treatment^35,36^. Total number of viable cells present in control group (cells without any treatment) was considered as 100% and accordingly the percentage of viable cell count compared to control group were determined in other groups.

### Analysis of cell migration by wound-healing assay

To determine the individual or combined (synergistic) effects of HDAC1 & HDAC2 inhibition as well as DNMT1 knockdown on cell migration, MDA-MB-231 cells were seeded into 6-well plates and at appropriate confluence points, wound were made as a straight scratch using a sterile 200 μl pipette tip. Debris was removed by washing the cells with PBS and then with growth medium to smooth the edges of the scratch. TSA treatment was performed 6 h after transfection of DNMT1 siRNA, at this time (after TSA treatment) the first scratch image was taken to show the 0 h image. Similarly, pictures were taken to visualize cell migration after 24 h and the area of the wound covered with migrated cells was measured.

### Chromatin condensation assay

The chromatin condensation assay provides a convenient method to analyze late stage apoptosis microscopically. To observe the induction of apoptosis by individual or synergistic effects of DNMT1 silencing and HDAC1 & HDAC2 inhibition after 24 h treatment, MDA-MB-231 cells were washed once with PBS and stained with Hoechst 33,342 stain (1 mg/ml) for10 min at 37 °C. Compacted chromatin of apoptotic cells binds to more amounts of stain compared to the healthy cells, which differentiate between healthy and apoptotic cells with condensed nuclei. To determine condensed nuclei, 5 randomly selected fields with 30–40 nuclei were observed under Epifluorescent Microscope (Olympus IX71) at excitation wavelength of 355–366 nm, emission wavelength of 465–480 nm. Percentage of condensed nuclei with respect to total number of nuclei was calculated. As reported previously, three independent experiments were done to calculate P value and validate the results^34,36^.

### RNA extraction and quantitative analysis of gene expression by qRT-PCR

MDA-MB-231 cells were harvested and rinsed properly in PBS for two times. RNA was extracted by using Trireagent as per the manufactures instruction. cDNA was synthesized taking total 1 μg RNA per reaction with oligodT and RevertAid First strand c-DNA synthesis kit (Thermo Scientific) was used as per the manufactures protocol. To analyze the expression of genes, specific primer pairs (Table S1. Primer pairs) and SYBR Green JumpStart^TM^Taq Ready mix in Realplex4 eppendorf system were used for qRT-PCR. Reaction was started with hot start for 5 min at 95 °C for denaturation, followed by 40 cycles taking appropriate primer pairs (Table S1. Primer pairs). A final extension step at 72 °C for 7 min was followed after the final cycle to complete polymerization. The expression of each gene was normalized with GAPDH as internal control.

### SDS-PAGE and western blot analysis

After harvesting, MDA-MB-231 cells were lysed using cell lysis buffer containing protease inhibitor cocktail (Sigma) and total cellular proteins were extracted and centrifuged at 14,000 g for 15 min at 4 °C. The concentration of protein was quantified by Bradford method as described previously^38^. Equal amount of proteins (20 µg) from each sample were separated in SDS-PAGE (depending upon size of the desire protein, percentage of gel was determined) and electro blotted to PVDF/nitrocellulose membrane (Millipore) followed by proper electrophoresis. The membrane was incubated for blocking in 3% BSA with TBST for two hours at RT. The blots were then incubated in primary antibody for overnight at 4 °C and then incubated in suitable HRP-conjugated secondary antibody after proper washing. Immunoreactive proteins were developed and detected on X-Ray film using ECL kit (Thermo Scientific). Specific protein band was confirmed based on the protein marker and datasheet of the primary antibody manufacturer. After each experiment, the images of the X-Ray films (blots) were snapped and desired area of the blots were cropped and saved for further analysis. Band intensity was quantified and normalized with β-actin (as an internal control) using the Gel documentation system and analyzed by Alpha imager software. The representative blot of replicates was presented in the Result Figures and the full-length original images (cropped) of representative blots were presented in the supplementary file.

### Nuclear protein preparation

Nuclear protein was prepared as described previously^38^. Briefly, after harvesting cells were washed twice in ice-cold PBS. Pellet was resuspended in 400 µl of ice cold hypotonic buffer [10 mM HEPES/KOH (pH 7.9), 2.0 mM MgCl2, 0.1 mM EDTA, 10 mM KCl, 1.0 mM DTT and 0.5 mM PMSF], mixed properly and kept on ice for 10 min. Then samples were vortexed properly and centrifuged at 15,000 g for 30 s at 4 °C. Nuclear pellet was collected, resuspended in 100 µl of ice-cold saline buffer [50 mM HEPES/KOH (pH 7.9), 50 mM KCl, 300 mM NaCl, 0.1 mM EDTA, 10% glycerol, 1.0 mM DTT and 0.5 mM PMSF] mixed properly and kept on ice for 30 min. Then samples were vortexed properly and centrifuged at 15,000 g for 5 min at 4 °C. Supernatant containing nuclear protein was collected and quantitated by Bradford method. Nuclear proteins were stored in aliquots at − 80 °C for further use or used directly. Equal amount of protein of each sample was separated by 10% SDS-PAGE and silver stained to check protein integrity and equal loading.

### Immunofluorescence staining

MDA-MB-231 ceils were seeded on glass cover slips in a 6-well culture plate for Immunofluorescence staining. After proper treatment cover slips with attached cells were taken and washed for three times with PBS. Cells were fixed with 80% ice cold methanol and were permeabilized with 0.25% Triton X-100. After PBS wash, cells were incubated in blocking solution (3% BSA in PBST) for 30 min, followed by incubation of primary antibody for overnight at 4 °C. After PBS wash, cells were incubated with suitable fluorescent tagged secondary antibody for 2 h at room temperature followed by PBS wash. Then cells were counterstained with DAPI immersed in an antifade solution and overlaid with a cover slip. Immunofluorescent proteins were visualized, detected and photographed by using Laser scanning confocal microscope (Leica).

### Microtubule regrowth assay (analysis of the colocalizations of γ-tubulin and TUBGCP2 complexes in mitotic cells)

After transfection of DNMT1 siRNA followed by 24 h of TSA treatment, cells were incubated in ice-cold DMEM containing 10% FBS for 30 min to depolymerize all cellular microtubules, and then medium was subsequently exchanged with pre-warmed (37 °C) growth medium. Then after 30 min cells were washed and fixed using ice cold methanol and were permeablized with 0.25% Triton X-100. Further, cells were immunostained and imaged using anti-γ-tubulin and anti-TUBGCP2 anibody. Mitotic cells were observed based on colocalizations of γ-tubulin and TUBGCP2 complexes through Laser scanning confocal microscope (Leica) after incubated with fluorescence labelled appropriate secondary antibodies and counterstained with DAPI immersed in an antifade solution.

### Statistical analysis

As described previously, statistical analysis of data was performed by SPSS software using one-way ANOVA followed by Tucky’s test^38^. Values of all the experiments observed in this study were presented as mean ± S.E.M. obtained from three different sets of experiments, *p* < 0.05 was considered as statistically significant (95% confidence interval) compared to control group (#), TSA treated group (δ) and DNMT1 siRNA transfected group (θ).

## Results

### Higher expression of centrosome proteins correlates with poor overall survival of breast cancer patients

**Figure 1:**
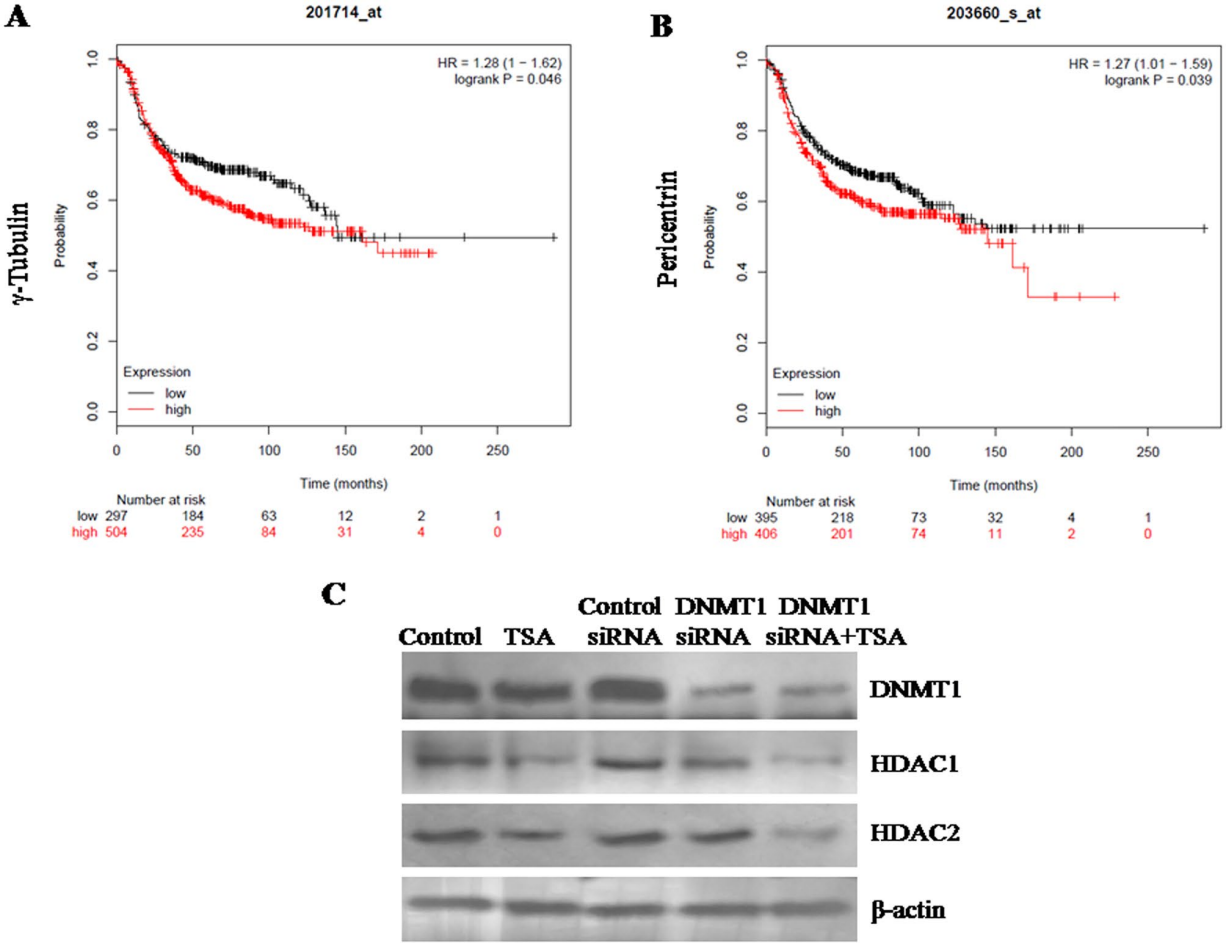
Overall survival analysis of patients using Kaplan–Meier survival plotter showing higher expression of (**A**) γ-tubulin (*p* = 0.046, HR = 1.28) and (**B**) pericentrine (*p* = 0.039, HR = 1.27) are associated with poor survival of breast cancer patients. Western blot analysis of the expression of DNMT1 as well as HDAC1 & HDAC2 showing (**C**) confirmation of the knock down of DNMT1 and inhibition of HDAC1 & HDAC2 after siRNA and TSA treatment respectively. The representative blots of replicates were presented here. The full-length original images (cropped) of representative blots were presented in the supplementary Fig. S2 of Supplementary information file.

To study the effect of change in expression of centrosome proteins (with vital role in centrosome amplification) γ-tubulin and pericentrine on overall survival of breast cancer patients, in silico analysis was performed based on publicly available database mining, as described previously^35,36^. Kaplan–Meier survival plotter was retrieved for free survival analysis (Fig. 1A,B). Elevated expression of γ-tubulin (*p* = 0.046, HR = 1.28) and pericentrine (*p* = 0.039, HR = 1.27) were observed to be associated with poor survival of breast cancer patients.

### Confirmation of the siRNA-mediated silencing of DNMT1 and inhibition of HDAC1 & HDAC2 by TSA treatment

Western analysis exhibited that DNMT1 expression was observed at negligible (significantly reduced) levels after DNMT1 siRNA transfection compared to control siRNA transfected cells. Successful inhibition of DNMT1 protein expression via transfection of DNMT1 siRNA had been reported previously^34,35^. Similarly, expression of HDAC1 and HDAC2 was observed to be reduced significantly after TSA treatment, as reported previously^36,37^. β-actin was used as loading control (Fig. 1C).

### Knockdown of DNMT1 and inhibition of HDAC1 & HDAC2 synergistically inhibit expression of centrosome protein γ-tubulin

**Figure 2:**
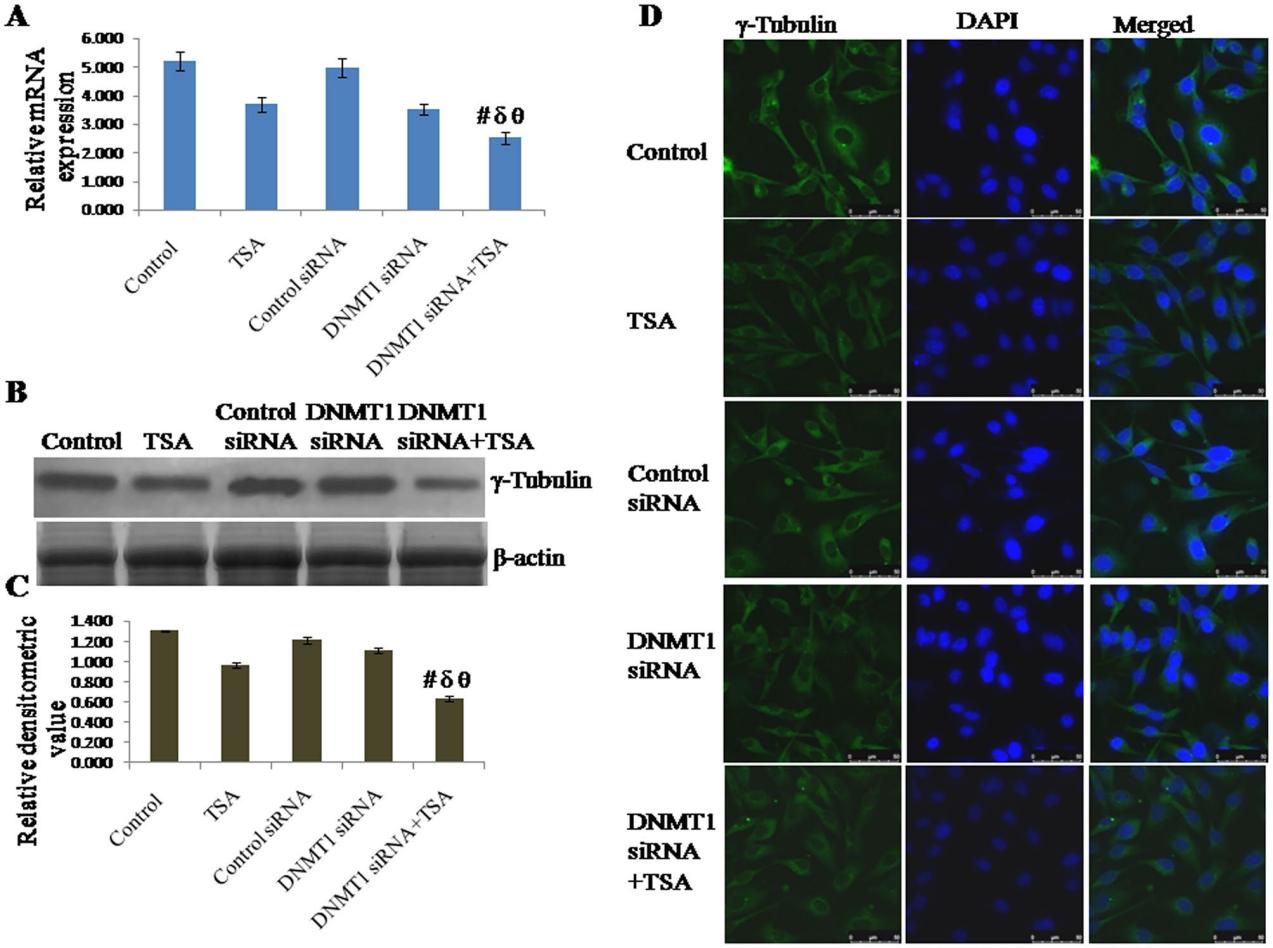
Expression of γ-Tubulin analyzed by (**A**) qRT-PCR using GAPDH as internal control and expressed in terms of relative mRNA expression, (**B**, **C**) Western blotting using β-actin as internal control and expressed in terms of relative densitometric value, (**D**) immunofluorescent staining and confocal microscopy to observe cellular localization of γ-Tubulin. The representative blots of replicates were presented here. The full-length original images (cropped) of representative blots were presented in the supplementary Fig. S3 of Supplementary information file. Data was represented as mean ± SEM., n = 3, *p* < 0.05. #, δ and θ represent; compared to control group, TSA treated group and DNMT1 siRNA transfected group respectively.

The mRNA expression of γ-tubulin was found to be down regulated by knockdown of DNMT1 as well as by specific inhibition of HDAC1 & HDAC2. However, mRNA expression of γ-tubulin was observed to be significantly down regulated due to synergistic effect of the knockdown of DNMT1 and specific inhibition of HDAC1 & HDAC2 compared to either knockdown of DNMT1 or inhibition of HDAC1 & HDAC2 (Fig. 2A). mRNA expression of γ-tubulin in cells with silencing of DNMT1 and specific inhibition of HDAC1 & HDAC2 was observed to be decreased to approximately 51% of control (untreated) cells, 31% of TSA treated cells, 50% of control siRNA treated cells and 28% of DNMT1 siRNA treated cells respectively. The protein level of γ-tubulin also follows similar variation pattern of its expression like mRNA (Fig. 2B,C). Protein level of γ-tubulin in cells with both knockdown of DNMT1 and specific inhibition of HDAC1 & HDAC2 was observed to be decreased to approximately 50% of control (untreated) cells, 35% of TSA treated cells, 49% of control siRNA transfected cells and 42% of DNMT1 siRNA transfected cells respectively. In addition, protein level of γ-tubulin was also observed in terms of cellular localization, which was observed to be similar like transcript and protein level (Fig. 2D).

### Knockdown of DNMT1 and inhibition of HDAC synergistically inhibit expression of centrosome protein TUBGCP2

**Figure 3:**
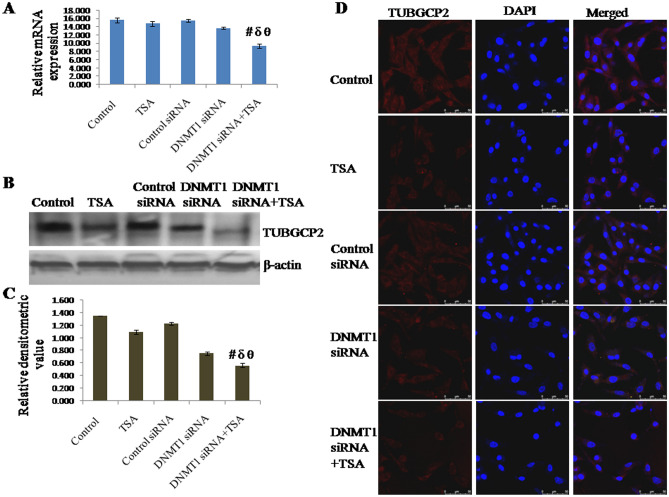
Expression of TUBGCP2 analyzed by (**A**) qRT-PCR using GAPDH as internal control and expressed in terms of relative mRNA expression, (**B**, **C**) Western blotting using β-actin as internal control and expressed in terms of relative densitometric value, (**D**) immunofluorescent staining and confocal microscopy to observe cellular localization of TUBGCP2. The representative blots of replicates were presented here. The full-length original images (cropped) of representative blots were presented in the supplementary Fig. S4 of Supplementary information file. Data was represented as mean ± SEM., n = 3, *p* < 0.05. #, δ and θ represent; compared to control group, TSA treated group and DNMT1 siRNA transfected group respectively.

The mRNA expression of TUBGCP2 was found to be down regulated by silencing DNMT1 as well as by specific inhibition of HDAC1 & HDAC2. However, mRNA expression of TUBGCP2 was observed to be significantly down regulated due to synergistic knockdown of DNMT1 and specific inhibition of HDAC1 & HDAC2 compared to either knockdown of DNMT1 or inhibition of HDAC1 & HDAC2 (Fig. 3A). mRNA expression of TUBGCP2 in cells with DNMT1 siRNA and TSA treatment was observed to be decreased to approximately 40% of control (untreated) cells, 36% of TSA treated cells, 39% of control siRNA treated cells and 31% of DNMT1 siRNA treated cells respectively (Fig. 3B,C). The protein level of TUBGCP2 also follows similar variation pattern of its expression like mRNA. Protein level of TUBGCP2 in cells with DNMT1 siRNA transfected and TSA treated was observed to be decreased to approximately 58% of control (untreated) cells, 49% of TSA treated cells, 56% of control siRNA transfected cells and 25% of DNMT1 siRNA transfected cells respectively. Surprisingly, here it was observed that epigenetic alterations not only modulated the expression of TUBGCP2 at transcript level but modulated at protein level also. In addition, protein level of TUBGCP2 was also observed in terms of cellular localization, which was observed to be similar like transcript and protein level (Fig. 3D).

### Knockdown of DNMT1 and inhibition of HDAC synergistically inhibit expression of centrosome protein pericentrin

**Figure 4:**
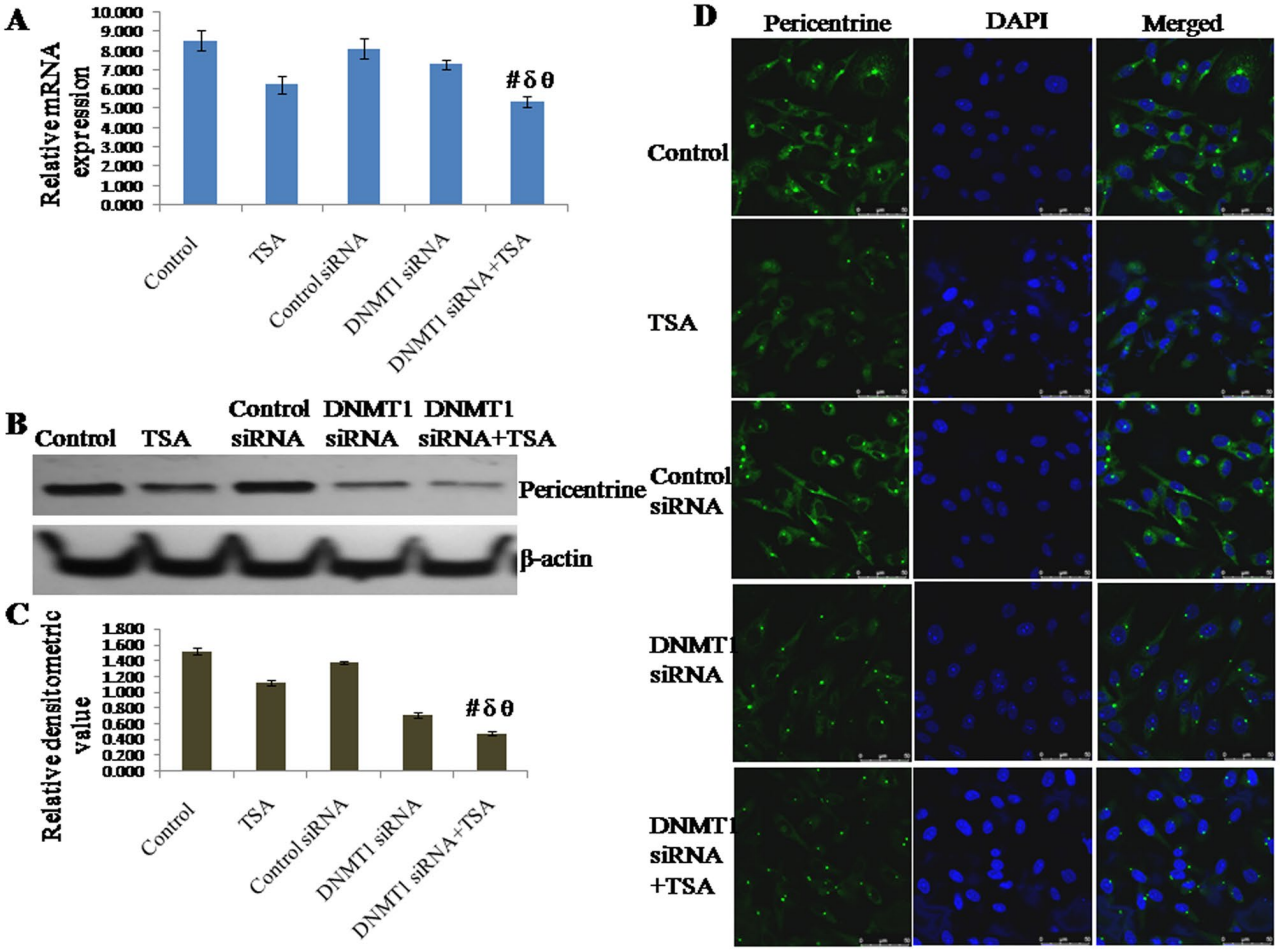
Expression of pericentrine analyzed by (**A**) qRT-PCR using GAPDH as internal control and expressed in terms of relative mRNA expression, (**B**, **C**) Western blotting using β-actin as internal control and expressed in terms of relative densitometric value, (**D**) immunofluorescent staining and confocal microscopy to observe cellular localization of pericentrin. The representative blots of replicates were presented here. The full-length original images (cropped) of representative blots were presented in the supplementary Fig. S5 of Supplementary information file. Data was represented as mean ± SEM., n = 3, *p* < 0.05. #, δ and θ represent; compared to control group, TSA treated group and DNMT1 siRNA transfected group respectively.

The expression of pericentrin at transcriptional level was found to be down regulated by silencing DNMT1 as well as by specific inhibition of HDAC1 & HDAC2. However, mRNA expression of pericentrin was observed to be significantly down regulated due to synergistic knockdown of DNMT1 and specific inhibition of HDAC1 & HDAC2 compared to either knockdown of DNMT1 or inhibition of HDAC1 & HDAC2 (Fig. 4A). mRNA expression of pericentrin in cells with DNMT1 siRNA and TSA treatment was observed to be decreased to approximately 37% of control (untreated) cells, 14% of TSA treated cells, 35% of control siRNA transfected cells and 26% of DNMT1 siRNA transfected cells respectively. The protein level of pericentrin also follows similar variation pattern of its expression like mRNA (Fig. 4B,C). Protein level of pericentrin in cells with DNMT1 siRNA transfected and TSA treated was observed to be decreased to approximately 68% of control (untreated) cells, 57% of TSA treated cells, 66% of control siRNA transfected cells and 33% of DNMT1 siRNA transfected cells respectively. Like TUBGCP2, it was also observed that epigenetic alterations modulate the expression of pericentrin both at transcript level and protein level. In addition, protein level of pericentrin was also observed in terms of cellular localization, which was observed to be similar like transcript and protein level (Fig. 4D).

### Knockdown of DNMT1 and inhibition of HDAC synergistically inhibit expression of α-tubulin

**Figure 5:**
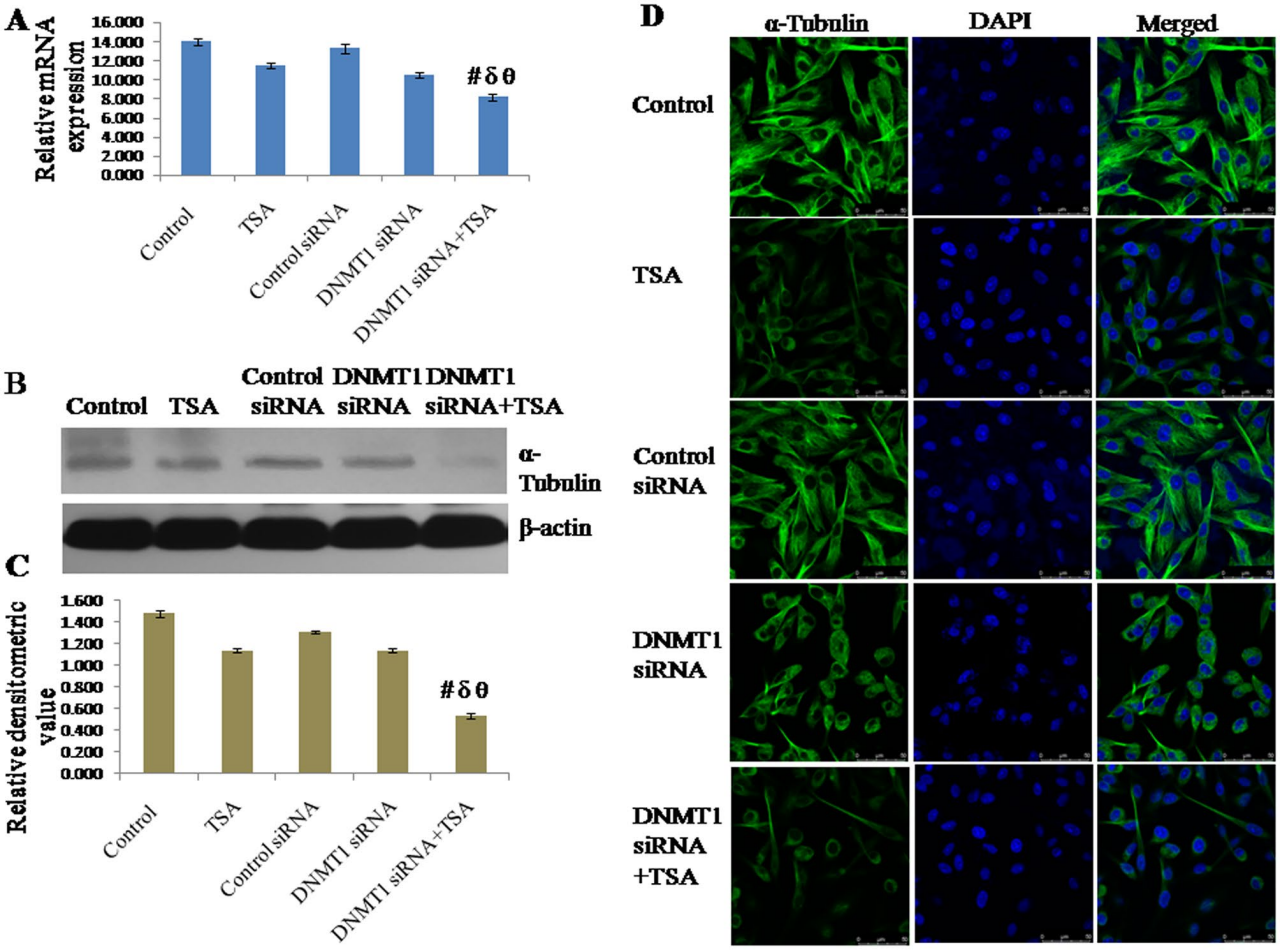
Expression of α-Tubulin analyzed by (**A**) qRT-PCR using GAPDH as internal control and expressed in terms of relative mRNA expression, (**B**, **C**) Western blotting using β-actin as internal control and expressed in terms of relative densitometric value, (**D**) immune fluorescent staining and confocal microscopy to observe cellular localization. The representative blots of replicates were presented here. The full-length original images (cropped) of representative blots were presented in the supplementary Fig. S6 of Supplementary information file. Data was represented as mean ± SEM., n = 3, *p* < 0.05. #, δ and θ represent; compared to control group, TSA treated group and DNMT1 siRNA transfected group respectively.

The expression of α-tubulin was analyzed here, as α-tubulin is found in the centrosome and participates to build microtubules together with β-tubulin, and expression of α-tubulin in atypical ductal hyperplasia and breast carcinoma increased significantly^12^. The mRNA expression of α-tubulin was found to be down regulated by silencing of DNMT1 as well as by specific inhibition of HDAC1 & HDAC2. However, mRNA expression of α-tubulin was observed to be significantly down regulated owing to synergistic knockdown of DNMT1 and specific inhibition of HDAC1 & HDAC2 compared to either knockdown of DNMT1 or inhibition of HDAC1 & HDAC2 (Fig. 5A). mRNA expression of α-tubulin in cells with DNMT1 knockdown and TSA treated was observed to be decreased to approximately 42% of control (untreated) cells, 30% of TSA treated cells, 38% of control siRNA transfected cells and 23% of DNMT1 siRNA transfected cells respectively. The protein level of α-tubulin also follows similar variation pattern of its expression like mRNA (Fig. 5B,C). Protein level of α-tubulin in cells with DNMT1 knockdown and TSA treatement was observed to be decreased to approximately 64% of control (untreated) cells, 54% of TSA treated cells, 58% of control siRNA transfected cells and 53% of DNMT1 siRNA transfected cells respectively. Like TUBGCP2 and pericentrin, here it was also observed that epigenetic alterations modulate expression of α-tubulin at mRNA level and protein level. In addition, protein level of α-tubulin was also observed in terms of cellular localization, which was observed to be similar like transcript level and protein level (Fig. 5D).

### Knockdown of DNMT1 and inhibition of HDAC synergistically inhibit co-localization of γ-tubulin and TUBGCP2 complex

**Figure 6:**
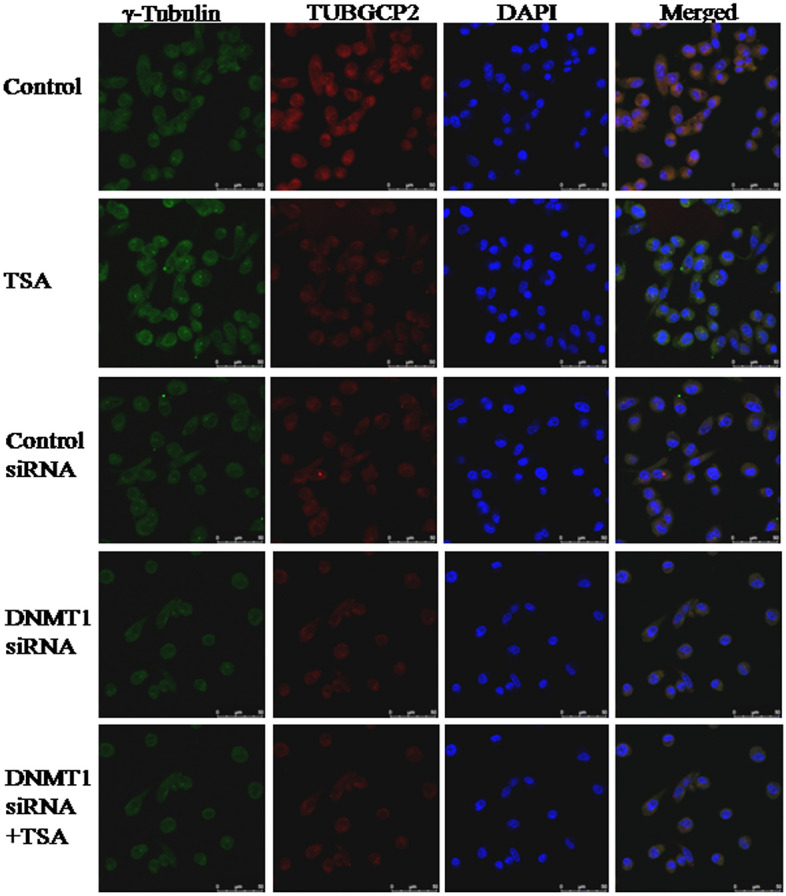
Immunofluorescent staining and confocal microscopy showing colocalizations of γ-tubulin and TUBGCP2 complexes in mitotic cells, which implicate knockdown of DNMT1 and specific inhibition of HDAC1 & HDAC2 synergistically, inhibit mitosis by reducing physical association of γ-tubulin with TUBGCP2, thereby inhibiting formation of γ-tubulin small complex (γTuSC). Ultimately, microtubule nucleation does not occur to form mitotic spindle for which less number of mitotic cells were observed.

As, centrosome takes active role in separation of chromosome during mitosis, microtubule nucleation occurs to form mitotic spindle. Therefore, γ-tubulin physically associates with TUBGCP2 to form γ-tubulin small complex (γTuSC). Analysis of the colocalization of γ-tubulin and TUBGCP2 complexes in mitotic cells suggest that, knockdown of DNMT1 and specific inhibition of HDAC1 & HDAC2 synergistically reduced mitosis compared to either knockdown of DNMT1 or inhibition of HDAC1 & HDAC2 by TSA (Fig. 6).

### Knockdown of DNMT1 and specific inhibition of HDAC1 & HDAC2 synergistically inhibit cell proliferation of MDA-MB-231 TNBC cells

**Figure 7:**
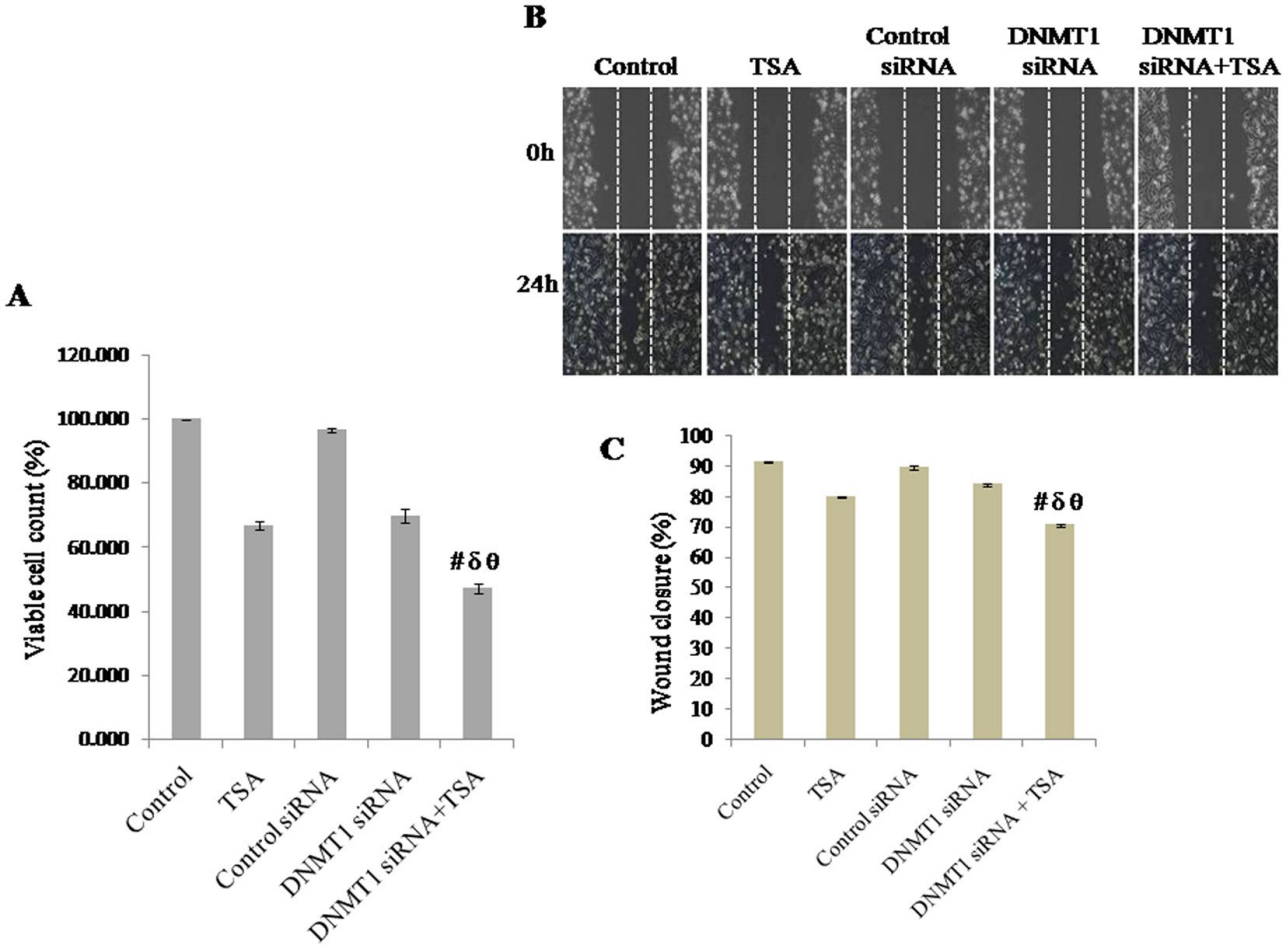
Effect of knockdown of DNMT1 and inhibition of HDAC1 & HDAC2, combined or due to either of any one on (**A**) cell viability analyzed by MTT assay and determined in terms of percentage of viable cells present, (**B**, **C**) cell migration, determined in terms of percentage of wound closure. Data was represented as mean ± SEM., n = 3, *p* < 0.05. #, δ and θ represent; compared to control group, TSA treated group and DNMT1 siRNA transfected group respectively.

The effect of silencing of DNMT1 and specific inhibition of HDAC1 & HDAC2 by TSA on proliferation of MDA-MB-231 TNBC cells were analyzed by MTT assay. Cell proliferation was observed to be significantly reduced by synergistic inhibition of HDAC1 & HDAC2 and knockdown of DNMT1 compared to either HDAC inhibition or knockdown of DNMT1. Viable cells after DNMT1 knockdown and TSA treatment was observed to be reduced to approximately 53% of control (untreated) cells, 30% of TSA treated cells, 52% of control siRNA transfected cells and 33% of DNMT1 siRNA transfected cells respectively (Fig. 7A).

### Knockdown of DNMT1 and inhibition of HDAC synergistically inhibit cell migration

Cellular migration being a property of metastasis, the synergistic effect of silencing of DNMT1 and specific inhibition of HDAC by TSA treatment was observed in MDA-MB-231 TNBC cells. Although, either silencing of DNMT1 or inhibition of HDAC1 & HDAC2 were able to reduce cell migration even after 24 h, both knockdown of DNMT1 and TSA treatment synergistically inhibited migration of MDA-MB-231 breast cancer cells significantly higher (Fig. 7B,C). Here, it was observed that approximate wound closure by control (untreated) cells was 91%, TSA treated cells was 80%, control siRNA transfected cells was 90%, DNMT1 siRNA transfected cells was 83%. However, migration of cells with both knockdown of DNMT1 and TSA treatment was significantly decreased, which shows approximate wound closure was 70% only.

### Silencing of DNMT1 and specific inhibition of HDAC1 & HDAC2 synergistically induces apoptosis in triple negative breast cancer cells

**Figure 8:**
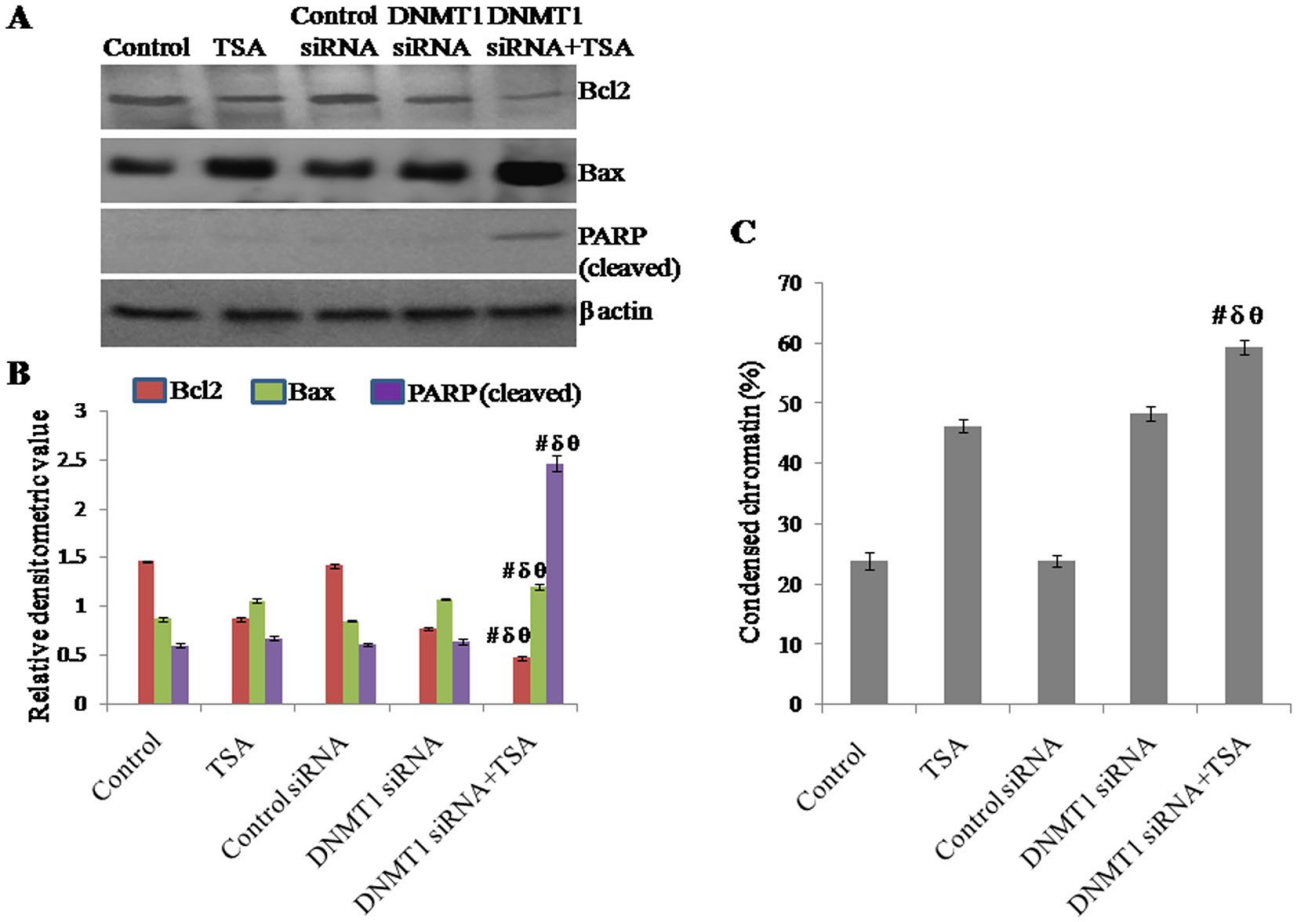
Effect of knockdown of DNMT1 and inhibition of HDAC1 & HDAC2, synergistically or due to either of any one on apoptosis observed in terms of (**A**, **B**) Western blotting analysis of Bcl2, Bax and PARP (cleaved) using β-actin as loading control and expressed in terms of relative densitometric value, (**C**) percentage of condensed chromatin with respect to total chromatin. The representative blots of replicates were presented here. The full-length original images (cropped) of representative blots were presented in the supplementary Fig. S7 of Supplementary information file. Data was represented as mean ± SEM., n = 3, *p* < 0.05. #, δ and θ represent; compared to control group, TSA treated group and DNMT1 siRNA transfected group respectively.

The impact of the knockdown of DNMT1 and specific inhibition of HDAC on apoptosis was determined by observing the apoptotic markers like Bcl2, Bax and PARP (cleaved) in protein level. The expression of Bcl2 was significantly down regulated and the expression of Bax and PARP (cleaved) were significantly upregulated because of synergistic outcome of silencing DNMT1 and inhibition of HDAC compared to either inhibition of HDAC or knockdown of DNMT1 (Fig. 8A,B). Protein level of Bcl2 in cells with DNMT1 knockdown and TSA treatement was observed to be reduced to approximately 68% of control (untreated) cells, 46% of TSA treated cells, 67% of control siRNA transfected cells and 39% of DNMT1 siRNA transfected cells respectively. Similarly, protein level of Bax in cells with DNMT1 knockdown and TSA treatment was observed to be approximately 1.385 times of control (untreated) cells, 1.135 times of TSA treated cells, 1.412 times of control siRNA transfected cells and 1.119 times of DNMT1 siRNA transfected cells respectively. Protein level of PARP (cleaved) in cells with DNMT1 knockdown and TSA treatment was observed to be approximately 4.11 times of control (untreated) cells, 3.653 times of TSA treated cells, 4.055 times of control siRNA transfected cells and 3.835 times of DNMT1 siRNA transfected cells respectively. Similarly, effect of the knockdown of DNMT1 and inhibition of HDAC on apoptosis was determined by observing condensed chromatins. The percentage of condensed chromatins in cells with DNMT1 knockdown and TSA treatment was observed to be approximately 2.47 times of control (untreated) cells, 1.28 times of TSA treated cells, 1.23 times of control siRNA transfected cells and 1.119 times of DNMT1 siRNA transfected cells respectively (Fig. 8C).

### DNMT1 plays a crucial role in cooperation with HDAC1 & HDAC2 in epithelial-mesenchymal transition in MDA-MB-231 breast cancer cells

EMT is the process by which epithelial cells lose cell polarity and cell–cell adhesion while simultaneously gaining mobility and invasiveness to become mesenchymal cells, and consequently EMT enables metastasis requiring invasiveness^39^. Therefore, the expression of E-cadherin, N-cadherin and vimentin; major proteins regulating EMT were analyzed. It was observed that DNMT1 plays a crucial role in cooperation with HDAC1 & HDAC2 in EMT. As, here it was found that silencing DNMT1 and inhibition of HDAC1 & HDAC2 synergistically induced the upregulation of E-cadherin whereas significantly down regulated the expression of N-cadherin and vimentin in terms of mRNA and protein level to inhibit EMT in MDA-MB-231 TNBC cells.

**Figure 9:**
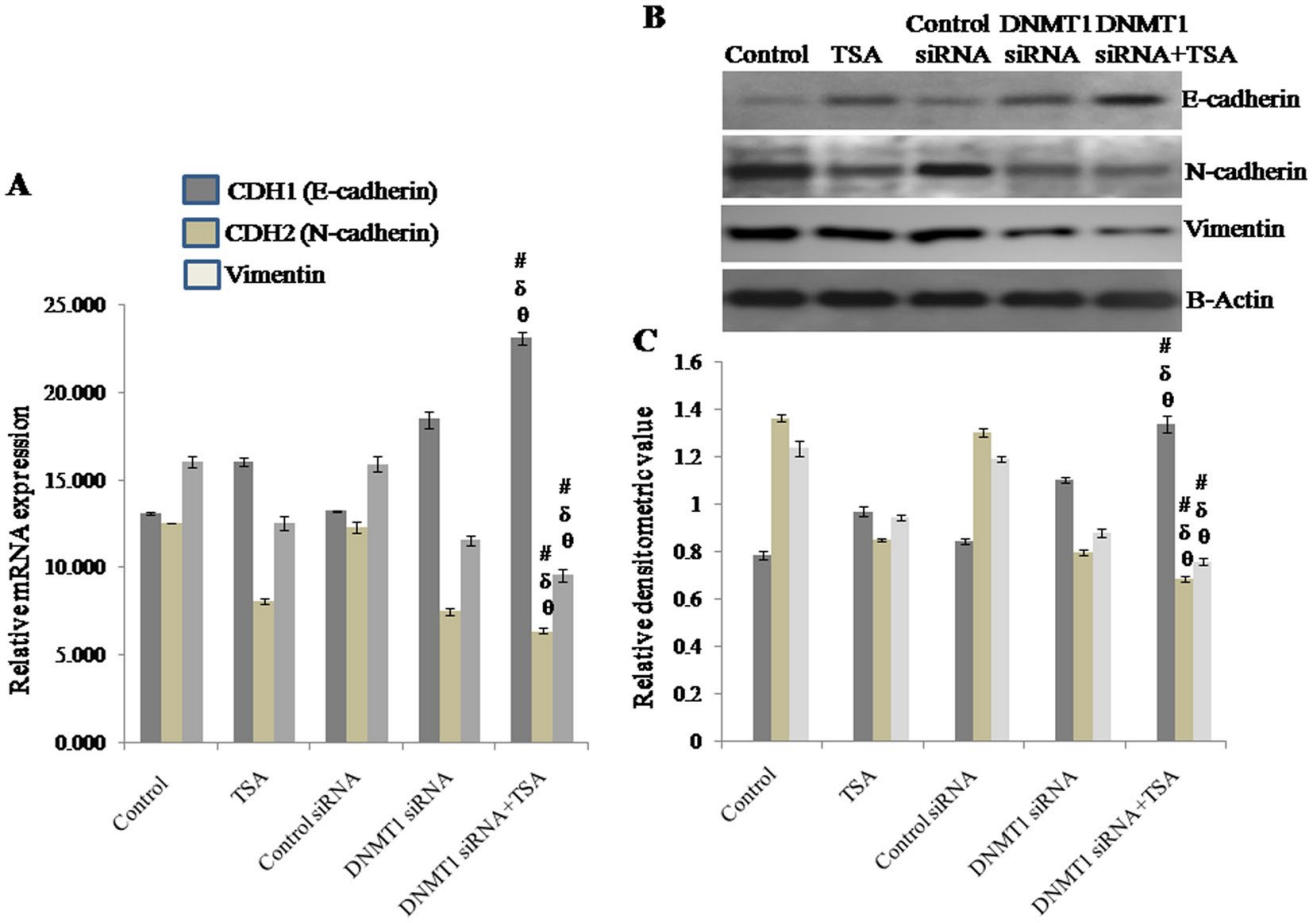
Expression of E-cadherin, N-cadherin and vimention analyzed by (**A**) qRT-PCR using GAPDH as internal control and expressed in terms of relative mRNA expression. Western blot analysis of E-cadherin, N-cadherin and vimention using β-actin as loading control, showing (**B**) effect of knockdown of DNMT1 and inhibition of HDAC1 & HDAC2 synergistically or due to either of any one on EMT, (**C**) relative densitometric value obtained from densitometric scanning after normalization with β-actin. The representative blots of replicates were presented here. The full-length original images (cropped) of representative blots were presented in the supplementary Fig. S8 of Supplementary information file. Data was represented as mean ± SEM., n = 3, *p* < 0.05. #, δ and θ represent; compared to control group, TSA treated group and DNMT1 siRNA transfected group respectively.

The mRNA expression of E-cadherin in cells with DNMT1 siRNA transfected and TSA treatment was observed to be approximately 1.765 times of control (untreated) cells, 1.437 times of TSA treated cells, 1.742 times of control siRNA transfected cells and 1.251 times of DNMT1 siRNA transfected cells respectively. Whereas, mRNA expression of N-cadherin in cells with DNMT1 knockdown and TSA treatment was observed to be reduced to approximately 49% of control (untreated) cells, 21% of TSA treated cells, 48% of control siRNA transfected cells and 15% of DNMT1 siRNA transfected cells respectively. Likewise, mRNA expression of vimentin in cells with DNMT1 knockdown and TSA treatment was observed to be decreased to approximately 40% of control (untreated) cells, 24% of TSA treated cells, 40% of control siRNA transfected cells and 17% of DNMT1 siRNA transfected cells respectively (Fig. 9A).

Similarly, protein level of E-cadherin in cells with DNMT1 knockdown and TSA treatment was observed to be approximately 1.7 times of control (untreated) cells, 1.38 times of TSA treated cells, 1.6 times of control siRNA transfected cells and 1.2 times of DNMT1 siRNA transfected cells respectively. Whereas, protein level of N-cadherin in cells with DNMT1 knockdown and TSA treatment was observed to be reduced to approximately 49% of control (untreated) cells, 19% of TSA treated cells, 47% of control siRNA transfected cells and 14% of DNMT1 siRNA transfected cells respectively. Protein level of vimentin in cells with DNMT1 knockdown and TSA treatment was observed to be decreased to approximately 38% of control (untreated) cells, 20% of TSA treated cells, 36% of control siRNA transfected cells and 14% of DNMT1 siRNA transfected cells respectively (Fig. 9B,C).

### Inhibition of oncogenic proteins and progression from G1 to S phase of the cell cycle owing to specific inhibition of HDAC and knockdown of DNMT1 in breast cancer cells

**Figure 10:**
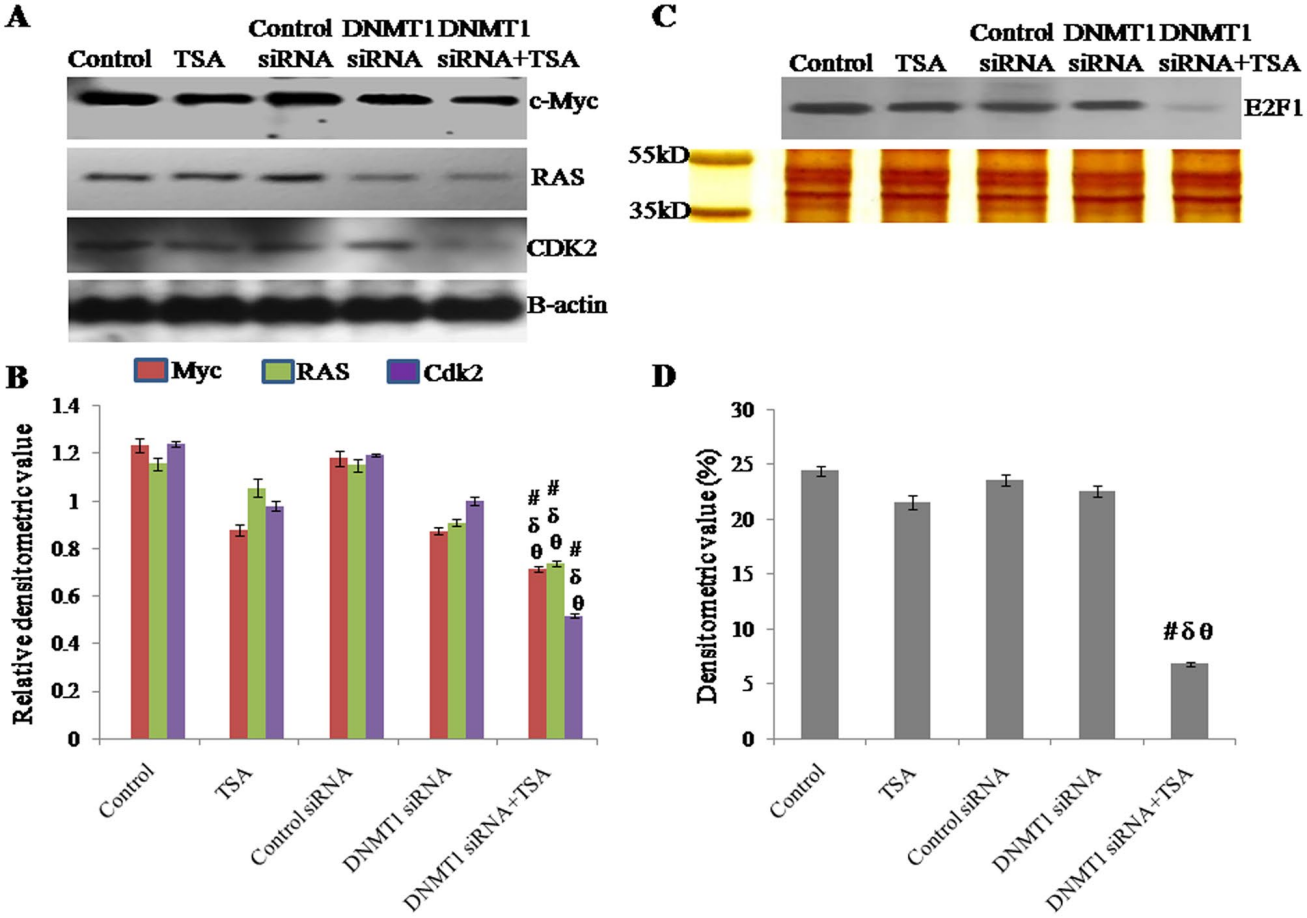
Protein level (expression) of (**A**) c-Myc, RAS and CDK2 analyzed by Western analysis using β-actin as loading control, (**B**) relative densitometric value obtained from densitometric scanning after normalization with β-actin. The representative blots of replicates were presented here. The full-length original images (cropped) of representative blots were presented in the supplementary Fig. S9 of Supplementary information file. Activation and nuclear translocation of (**C**) E2F1 analyzed by Western analysis of nuclear protein, and SDS-PAGE and silver staining of whole nuclear extract (protein) showing protein integrity and equal loading, (**D**) Densitometric value obtained from densitometric scanning. The representative blot of replicates was presented here. The full-length original images (cropped) of representative blot and silver stained gel were presented in the supplementary Fig. S10. Data was represented as mean ± SEM., n = 3, *p* < 0.05. #, δ and θ represent; compared to control group, TSA treated group and DNMT1 siRNA transfected group respectively.

Myc and RAS cooperatively regulate activation of G1 Cdk activity as well as induce accumulation of active cyclin E/Cdk2 and E2F^25^. Therefore, expressions of cMyc and RAS as well as Cdk2 were analyzed by western analysis. Besides, the effect of silencing of DNMT1 and specific inhibition of HDAC1 & HDAC2 on activation and nuclear translocation of E2F1 was checked. Here it was observed that the expression of cMyc, RAS and Cdk2 were significantly down regulated due to synergistic inhibition of HDAC1 & HDAC2 and silencing DNMT1 compared to either inhibition of HDAC or silencing of DNMT1. Protein level of cMyc in cells with DNMT1 knockdown and TSA treatment was observed to be reduced to approximately 41% of control (untreated) cells, 18% of TSA treated cells, 39% of control siRNA transfected cells and 18% of DNMT1 siRNA transfected cells respectively. Protein level of RAS in cells with DNMT1 knockdown and TSA treatment was observed to be reduced to approximately 36% of control (untreated) cells, 30% of TSA treated cells, 35% of control siRNA transfected cells and 19% of DNMT1 siRNA transfected cells respectively. Whereas, protein level of Cdk2 in cells with DNMT1 knockdown and TSA treatment was observed to be reduced to approximately 58% of control (untreated) cells, 47% of TSA treated cells, 56% of control siRNA transfected cells and 48% of DNMT1 siRNA transfected cells respectively. Thus, this result implicate that HDAC and DNMT1 cooperatively regulate cell proliferation through oncogene cMyc and RAS (Fig. 10A,B).

The effect of silencing DNMT1 and/or inhibition of HDAC1 and HDAC2 on activation and nuclear translocation of E2F1 was analyzed based on the nuclear protein level of E2F1. Nuclear translocation of E2F1 was significantly down regulated due to synergistic inhibition of HDAC1 & HDAC2 and silencing DNMT1 compared to either inhibition of HDAC or silencing of DNMT1. Nuclear level of E2F1 in cells with DNMT1 knockdown and TSA treated was observed to be reduced to approximately 72% of control (untreated) cells, 47% of TSA treated cells, 73% of control siRNA transfected cells and 69% of DNMT1 siRNA transfected cells respectively (Fig. 10C,D).

Considering all the parameters studied here, epigenetic alterations produced due to synergistic inhibition of HDAC1 & HDAC2 by TSA and silencing of DNMT1 is significantly effective compared to either inhibition of HDAC1 & HDAC2 or silencing of DNMT1.

## Discussion

There are limited targeted treatment options for TNBC. However, various approaches have been employed to prevent the progression of such dreadful disease. This study emphasized specific role of epigenetic modifiers like DNMT1 and HDAC1 & HDAC2 on proliferation and apoptosis, as well as aggressiveness, invasion and metastasis in terms of EMT through centrosome amplification in breast cancer. Centrosome amplification is a common characteristic of tumor cells with existence of the mechanisms to cluster excess centrosomes that ensure successful division. Inhibition of centrosome clustering represents a promising tumor-cell-selective therapeutic approach^40^. TNBC is histologically highly aggressive with high recurrence, metastasis and mortality rates. TNBC and non-TNBC follow different metastasis progression motifs, but both form novel test beds, allowing the severity and nature of centrosome amplification to distinguish them apart^27^. Even so, centrosome amplification is associated with overexpression of centrosome proteins^11,12,15^. Higher expressions of α-tubulin and γ-tubulin have been reported in breast carcinomas. In addition, breast cancer cell lines derived from TNBC patients exhibit higher incidence and severity of centrosome amplification, elevated expression of centrosome proteins and centrosome amplification markers compared to cell lines derived from non-TNBC patients^27^. Upregulation of TUBGCP2 has been reported in the tumor tissues compared to the tumor-adjacent normal breast tissues^41^. Here it was observed that influence of the expression of centrosome proteins on overall survival of breast cancer patients analyzed through KM plotter indicates poor prognosis. Therefore, analysis of synergistic effects of silencing of DNMT1 and inhibition of HDAC1 & HDAC2 on γ-tubulin, TUBGCP2, and pericentrin expression at mRNA level, protein level and cellular distribution shows significant downregulation. Thus, in light of the expression of centrosome protein, results reflect significant inhibition of microtubule nucleation and centrosome amplification. As, microtubule nucleation in centrosomes is regulated by γ-tubulin via TuRCs^42^. In addition, significant downregulation of α-tubulin at mRNA level, protein level and cellular distribution reflects inhibition of proliferation, as higher expression of α-tubulin has been reported in atypical ductal hyperplasia and breast carcinoma^12^. Significant reduction of dividing cells due to synergistic effect of the DNMT1 silencing and inhibition of HDAC1 & HDAC2 further confirmed here inhibition of microtubule nucleation at the centrosome, assured based on co-localization of γ-tubulin and TUBGCP2 complexes.

Correlation of the function of certain genes with underlying cellular metabolites was interpreted by observing cell survival and migration pattern of MDA-MB-231 breast cancer cells after knockdown of DNMT1 and specific inhibition of HDAC1 & HDAC2. Overexpression of HDACs is reported in many cancer types including TNBC and HDAC inhibitors inhibit cell migration and proliferation^43^. DNMT1 is essential for maintenance of DNA methylation, proliferation, survival and facilitates metastasis in breast cancer, where as silencing of DNMT1 inhibits proliferation, migration and invasion abilities of breast cancer cells^44,45^. Surprisingly, here it was observed that silencing of DNMT1 and inhibition of HDAC1 & HDAC2 synergistically inhibited cellular migration and survival of MDA-MB-231 cells. Inhibition of cell proliferation and motility is related to cellular apoptosis, therefore the effect of inhibition of epigenetic modifiers DNMT1 and HDAC1 & HDAC2 on apoptotic markers like Bcl2, Bax and PARP (cleaved) as well as on chromatin condensation was observed. Increased HDAC expression is reported to induce cancer cell proliferation leading to poor prognosis in patients with various types of cancer, whereas HDAC inhibition leads to DNA damage, cell cycle arrest, and/or apoptosis^46,47^. Likewise, inhibition of DNMT1 expression induces apoptosis^48^. Dramatically, in this study it was observed that induction of apoptosis in MDA-MB-231 cells owing to synergistic silencing of DNMT1 and inhibition of HDAC1 & HDAC2, which further confirmed cooperative action of HDAC and DNMT1 in regulation of apoptosis in TNBC cells. Coordinated regulation of DNMT1 and HDAC is required for effective modulation of TNBC cell proliferation which is the cause of aggressive metastasis^49,50^. Metastasis, poor survival and increased risk of recurrence in breast cancer patients are contributed by EMT^51^. Cancer cells undergo EMT through lose of proper target recognition and activation of self-sufficient autocrine loops of growth signal-mechanisms to avoid apoptosis^52^. Expression of important members of the EMT like E-cadherin, N-cadherin and vimentin were analyzed here. This is because, E-cadherin-mediated loss of adhesions plays an important role in the metastasis of epithelial tumors from benign to invasive states and induces metastatic dissemination and activation of various EMT transcription factors^53^. N-cadherin is expressed in highly invasive cancer cells that do not express (or express poorly) E-cadherin^54^. Vimentin contributes to EMT by mediating cytoskeletal organization and focal adhesion maturation, as well as induces changes in cell shape, motility and adhesion^20,55^. Here, it was observed that knockdown of DNMT1 and specific inhibition of HDAC1 & HDAC2 synergistically induced the expression of E-cadherin as well as inhibited the expression of N-cadherin and vimentin. The results obtained here suggest that epigenetic modifiers like DNMT1 and HDAC regulate EMT and inhibition of EMT induces apoptosis in TNBC cells. This finding is supported by one of the earliest studies showing that inhibition of DNMT1 reduces breast cancer cell migration by enhancing E-cadherin expression through downregulation of Snail and Slug^36^. PCM of centrosome possess attachment sites for vimentin intermediate filaments, and lower vimentin level cannot form extended intermediate filaments but preferentially form perinuclear aggregate^21^. Thus, EMT appears to be facilitated by centrosome amplification in TNBC cells^56^. Since centrosome amplification is associated with overexpression of centrosome proteins, the impact of epigenetic alterations on centrosome amplification was demonstrated here in terms of expression of centrosome proteins^11,12,15^. Overexpression of pericentrin and TUBGCP2 are associated with aneuploidy and centrosome abnormalities^57^. Pericentrin plays an important role in cell division because it recruits γ-TuRCs to PCM^58^. However, centrosome aberrations have been reported to induce proliferation by ignoring microtubule-dependent tissue integrity as well as centrosome aberrations and chromosome instability enhance tumorigenesis and intratumoral heterogeneity in human breast cancer^59,60^. Thus, TNBC progression is driven by cell proliferation and centrosome amplification through activation of EMT. Therefore, findings of this study suggest that DNMT1 and HDAC cooperatively regulate EMT in TNBC cells through centrosome amplification, whereas silencing of DNMT1 and specific inhibition of HDAC1 and HDAC2 synergistically inhibits EMT through inhibition of centrosome amplification.

In addition, E-cadherin expression is down regulated by a posttranscriptional mechanism in cells expressing cMyc, as cMyc-induced cell transformation requires E-cadherin inhibition^61^. Myc has been reported to mediate stem cell-like cancer cells and EMT in TNBC, as well as Myc cooperates with Ras to induce the accumulation of active cyclins E/Cdk2 and E2F^24,25,62^. The role of E2F has been observed in tumour development and metastasis, specifically E2F1 direct metastasis of breast cancer cells by altering cell migration^63,64^. E2F regulates centrosome amplification and activators of E2F are reported to signal and maintain centrosome amplification in breast cancer cells^22^. On the other hand, Cdks are reported to regulate centrosome cycle and mediate oncogene-dependent centrosome amplification, as well as activation of Cdk2-cyclin A activity and E2F are required for centrosome division^65,66^. Therefore, in addition to the regulation of EMT by Myc, there exists a correlation between Myc and Ras in regulating E2F-mediated centrosome amplification supported by Cdks in breast cancer. Results obtained by analyzing the expression of cMyc, Ras and Cdk2, and the nuclear translocation of E2F1 showed that synergistic inhibition of HDAC and knockdown of DNMT1 decreased the expression of Myc, Ras and Cdk2, as well as inhibited the nuclear translocation of E2F1. In light of the role of HDAC, this finding is supported by studies showing that cMyc downregulation due to HDAC inhibition results in TRAIL activation and apoptosis^67^. Contrarily, low E2F1 expression strongly determines a positive outcome in breast cancer with low metastasis risk, further supporting the E2F1-related findings obtained here^68^. Thus, in TNBC, DNMT1 and HDAC cooperatively contribute key roles in the regulation of cell proliferation and apoptosis through Myc/Ras axis mediated centrosome amplification. Synergistic inhibition of HDAC and silencing of DNMT1 declined centrosome amplification, induced apoptosis and inhibited cell proliferation through inhibition of E2F1 nuclear translocation and down regulation of Myc and Ras.

**Figure 11:**
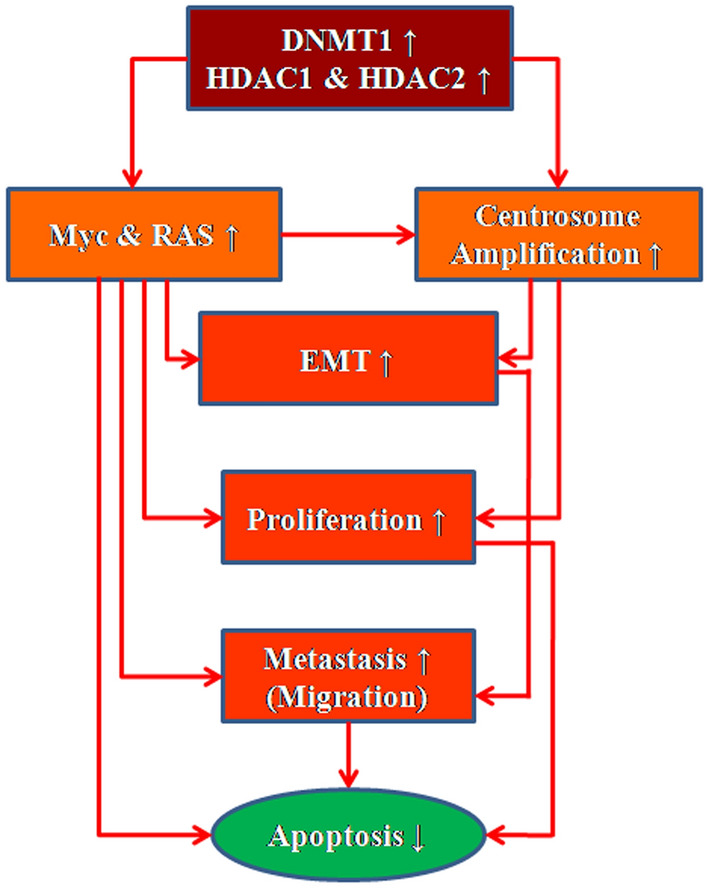
Schematic diagram showing DNA methylation and histone deacetylation-mediated chromatin remodelling modulate EMT via Myc/RAS pathway and centrosome amplification to regulate apoptosis in triple negative breast cancer cells.

Findings of this study concluded that, epigenetic alteration by siRNA-mediated silencing of DNA methylation and inhibition of histone deacetylation-mediated chromatin remodeling via specific inhibition of HDAC1 & HDAC2 synergistically inhibit cell proliferation and induce apoptosis by targeting EMT through modulation of centrosome amplification in triple negative human breast cancer cells (Fig. 11).

## Supplementary Information

Supplementary Information 1.

Supplementary Information 2.

Supplementary Information 3.

Supplementary Information 4.

Supplementary Information 5.

Supplementary Information 6.

Supplementary Information 7.

Supplementary Information 8.

Supplementary Information 9.

Supplementary Information 10.

Supplementary Information 11.

## Data Availability

Data is contained with the article or supplementary material.
