# Supplementary material for: Epigenetic alterations impede epithelial-mesenchymal transition by modulating centrosome amplification and Myc/RAS axis in triple negative breast cancer cells
Source: Sci Rep. 2023 Feb 11;13:2458. doi: 10.1038/s41598-023-29712-8 (PMC9922331; doi:10.1038/s41598-023-29712-8)
Supplement: Supplementary file 1 — Supplementary Information 1. [file 41598_2023_29712_MOESM1_ESM.docx]

Table S1: Primer pairs.

| Genes | Sequences of primer pairs (F: forward, R: reverse) |
| --- | --- |
| γ-tubulin | F: 5’-GACACATCCAGGGAGATTGTGC-3’  R: 5’-GCCAACCAGTAAGGCAGATGAG-3’ |
| TUBGCP2 | F: 5’-TGATGTTCGTGCCTCAGAGC-3’  R: 5’-TAGACCTCAGCCCCATCTCC-3’ |
| Pericentrin | F: 5’-GGTCGATCTCGTAGCTCAGG-3’  R: 5’-GTCAGGCTCTTCTCCGTCAC-3’ |
| α-tubulin | F: 5’-TCTTCCAGCTACCAAGAAACCG-3’  R: 5’-TTTTTCAGGGGGATACCTGGC-3’ |
| CDH1 (E-cadherin) | F 5’-CGAGAGCTACACGTTCACGG-3’  R 5’-GGGTGTCGAGGGAAAAATAGG-3’ |
| CDH2 (N-cadherin) | **F:** 5-CCCATACACCAGCCTGGAAC-3  **R**: 5-ACTAACCCGTCGTTGCTGTT-3 |
| Vimentin | **F:** 5-CCGCACATTCGAGCAAAGAC-3  **R:** 5-ATTCAAGTCTCAGCGGGCTC-3 |
