# Supplementary figures and images for: Epigenetic alterations impede epithelial-mesenchymal transition by modulating centrosome amplification and Myc/RAS axis in triple negative breast cancer cells
Source: Sci Rep. 2023 Feb 11;13:2458. doi: 10.1038/s41598-023-29712-8 (PMC9922331; doi:10.1038/s41598-023-29712-8)

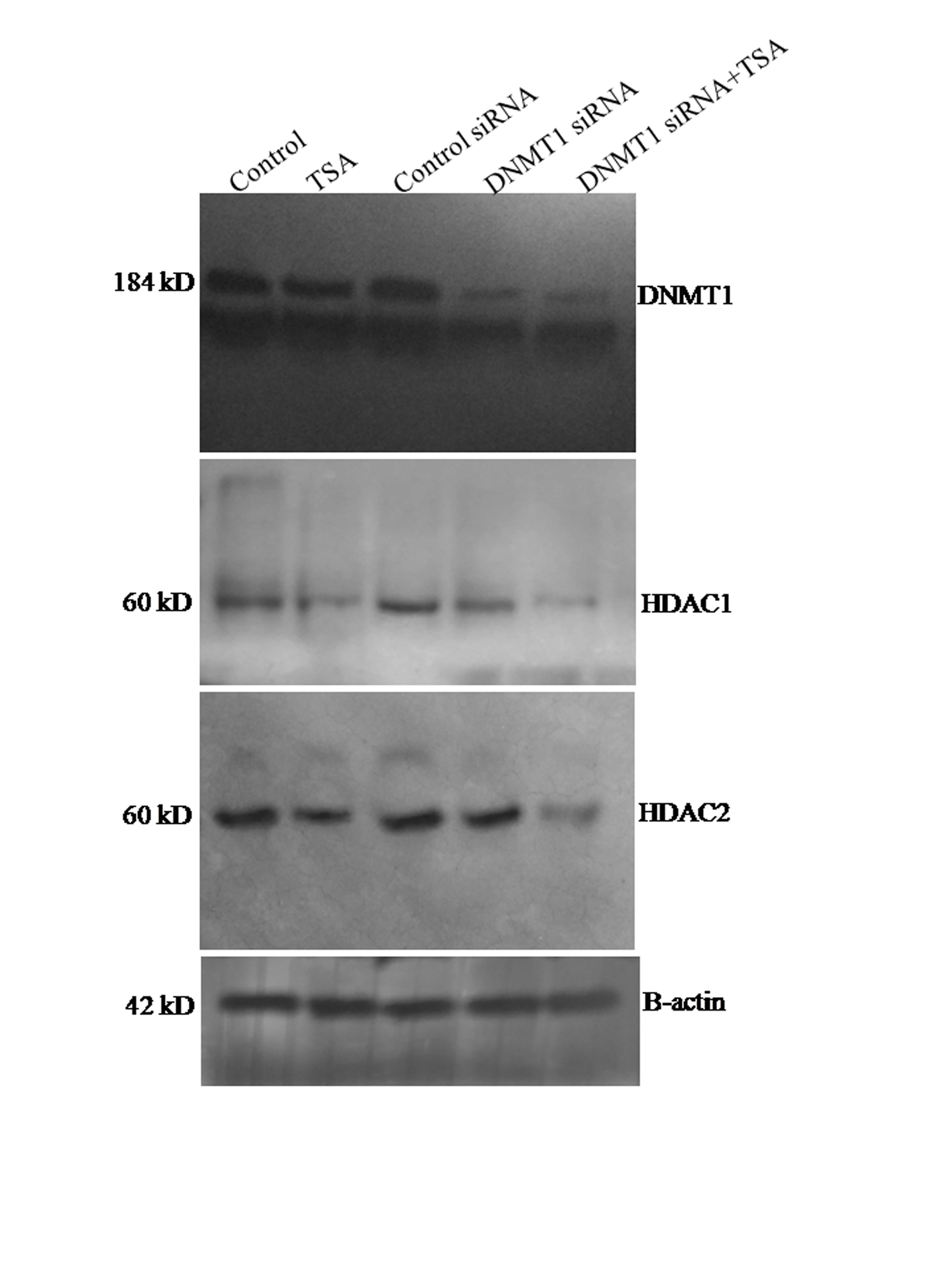

Supplement: Supplementary file 2 — Supplementary Information 2. [file 41598_2023_29712_MOESM2_ESM.tif]

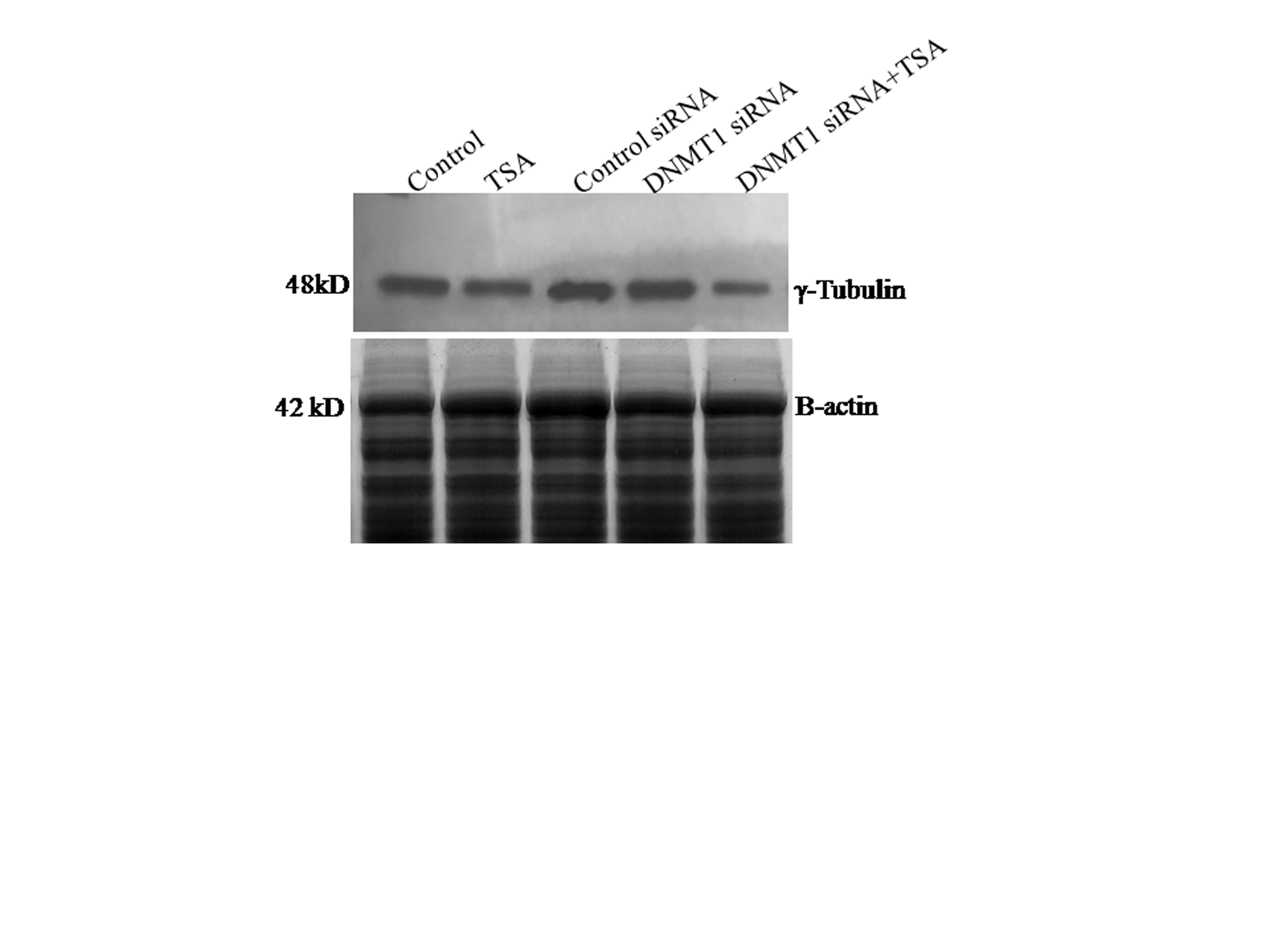

Supplement: Supplementary file 3 — Supplementary Information 3. [file 41598_2023_29712_MOESM3_ESM.tif]

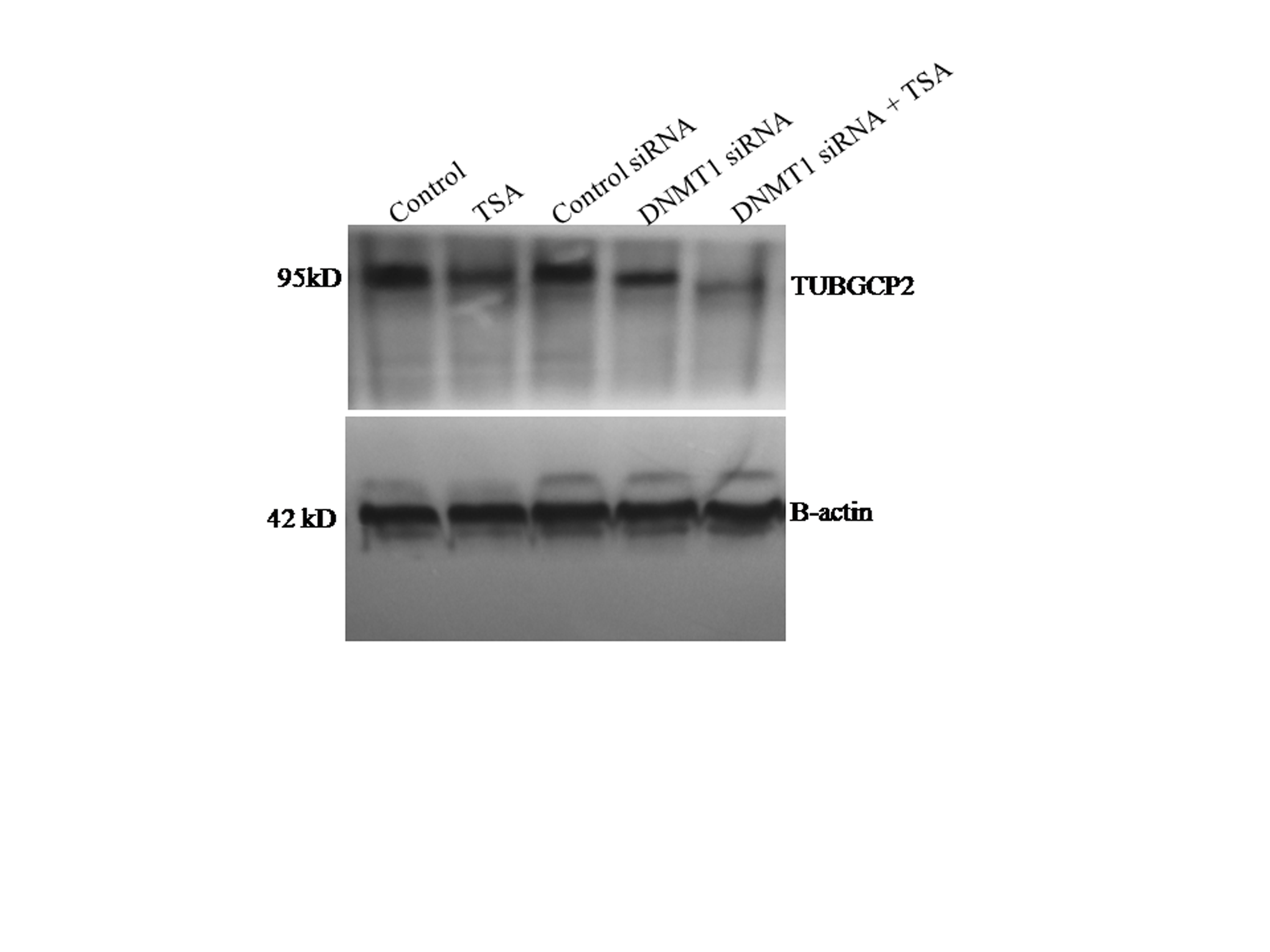

Supplement: Supplementary file 4 — Supplementary Information 4. [file 41598_2023_29712_MOESM4_ESM.tif]

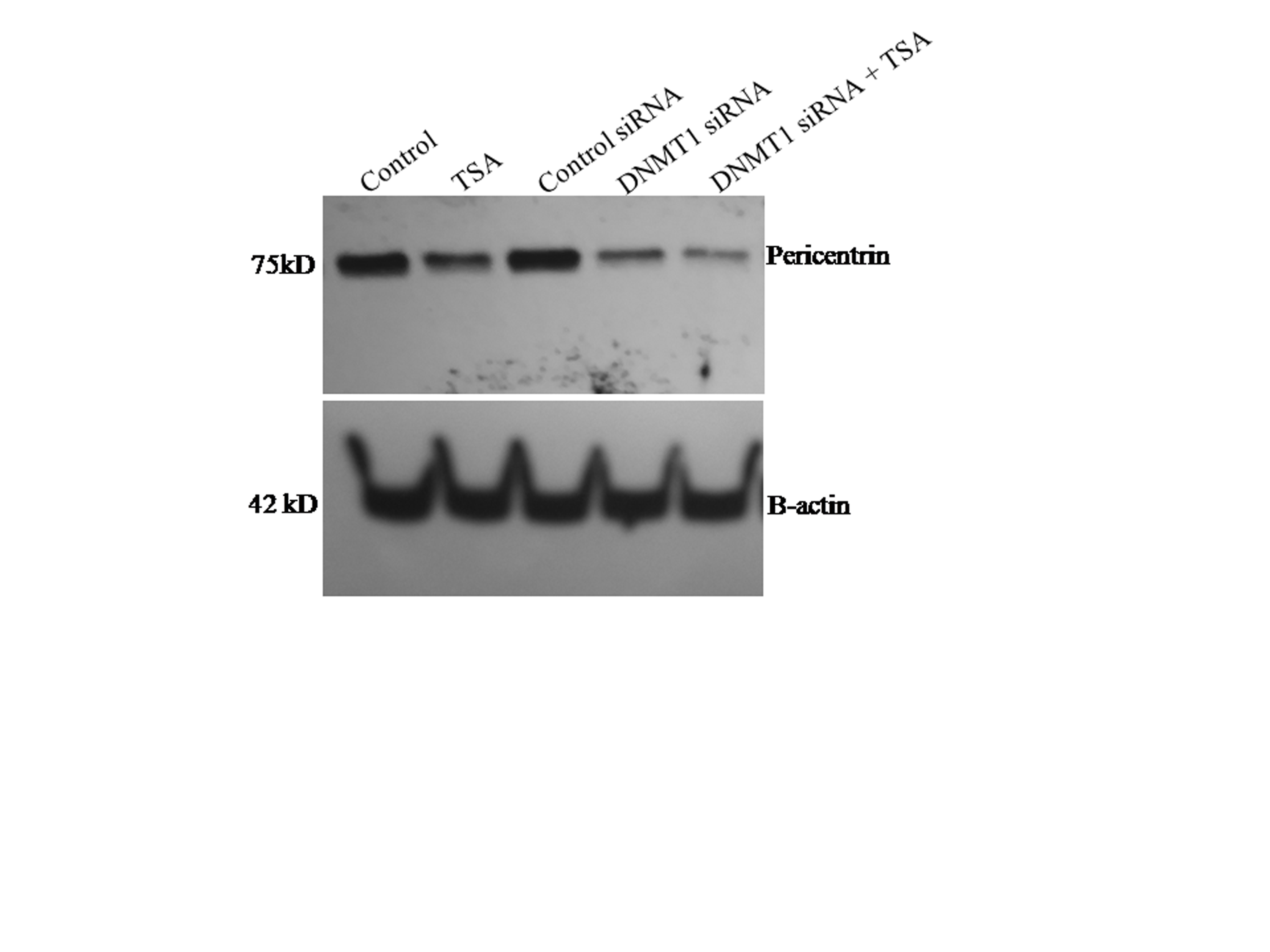

Supplement: Supplementary file 5 — Supplementary Information 5. [file 41598_2023_29712_MOESM5_ESM.tif]

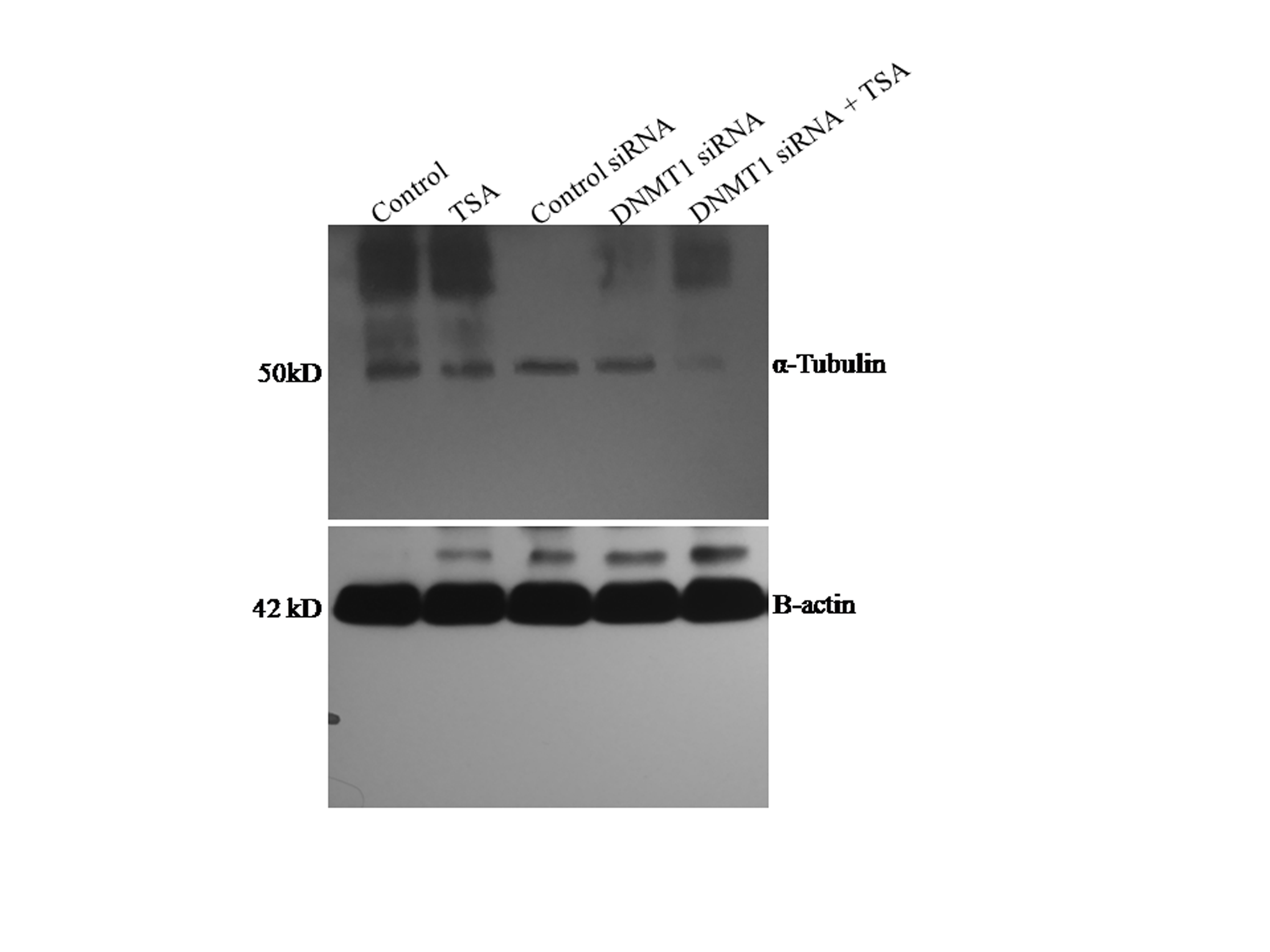

Supplement: Supplementary file 6 — Supplementary Information 6. [file 41598_2023_29712_MOESM6_ESM.tif]

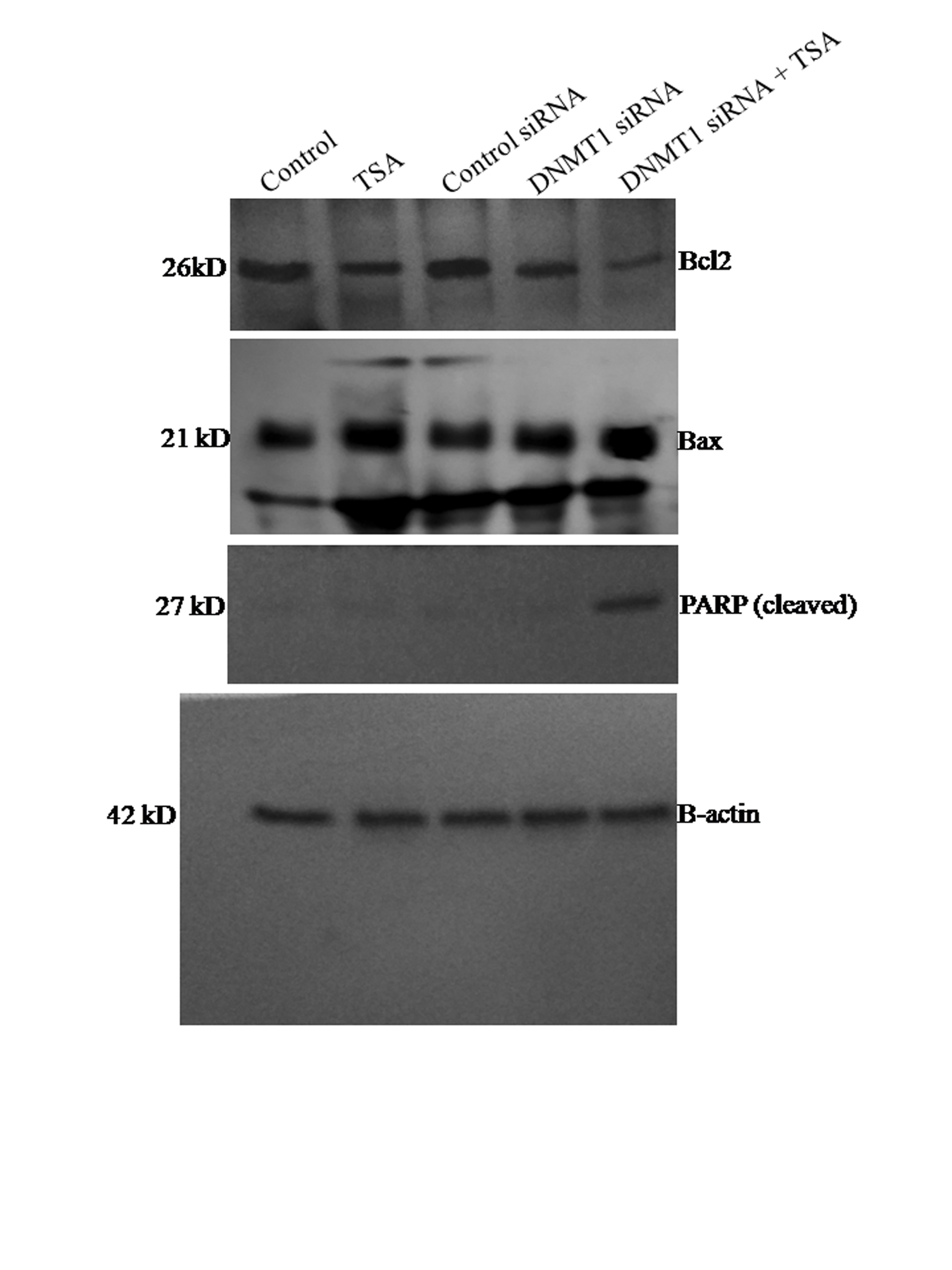

Supplement: Supplementary file 7 — Supplementary Information 7. [file 41598_2023_29712_MOESM7_ESM.tif]

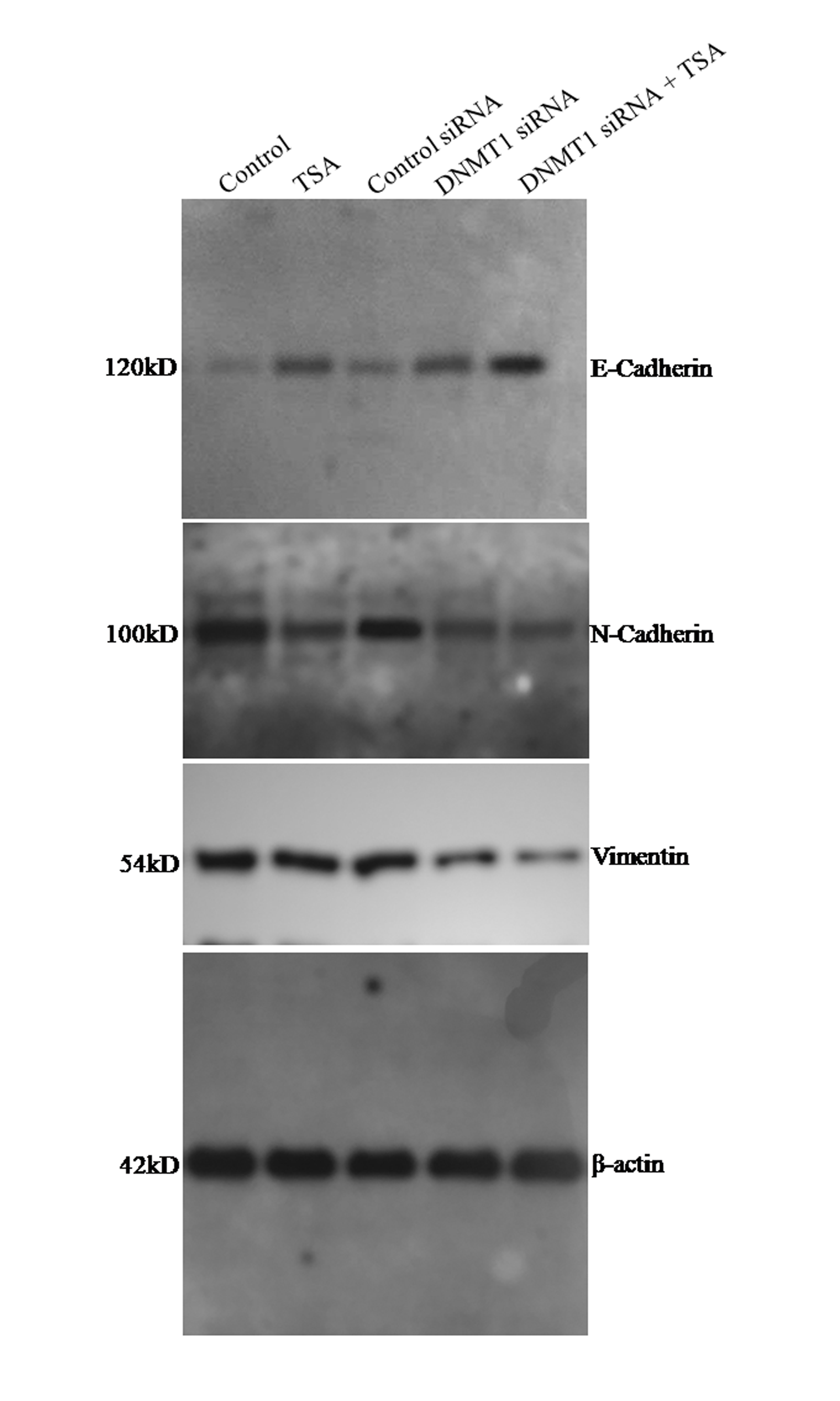

Supplement: Supplementary file 8 — Supplementary Information 8. [file 41598_2023_29712_MOESM8_ESM.tif]

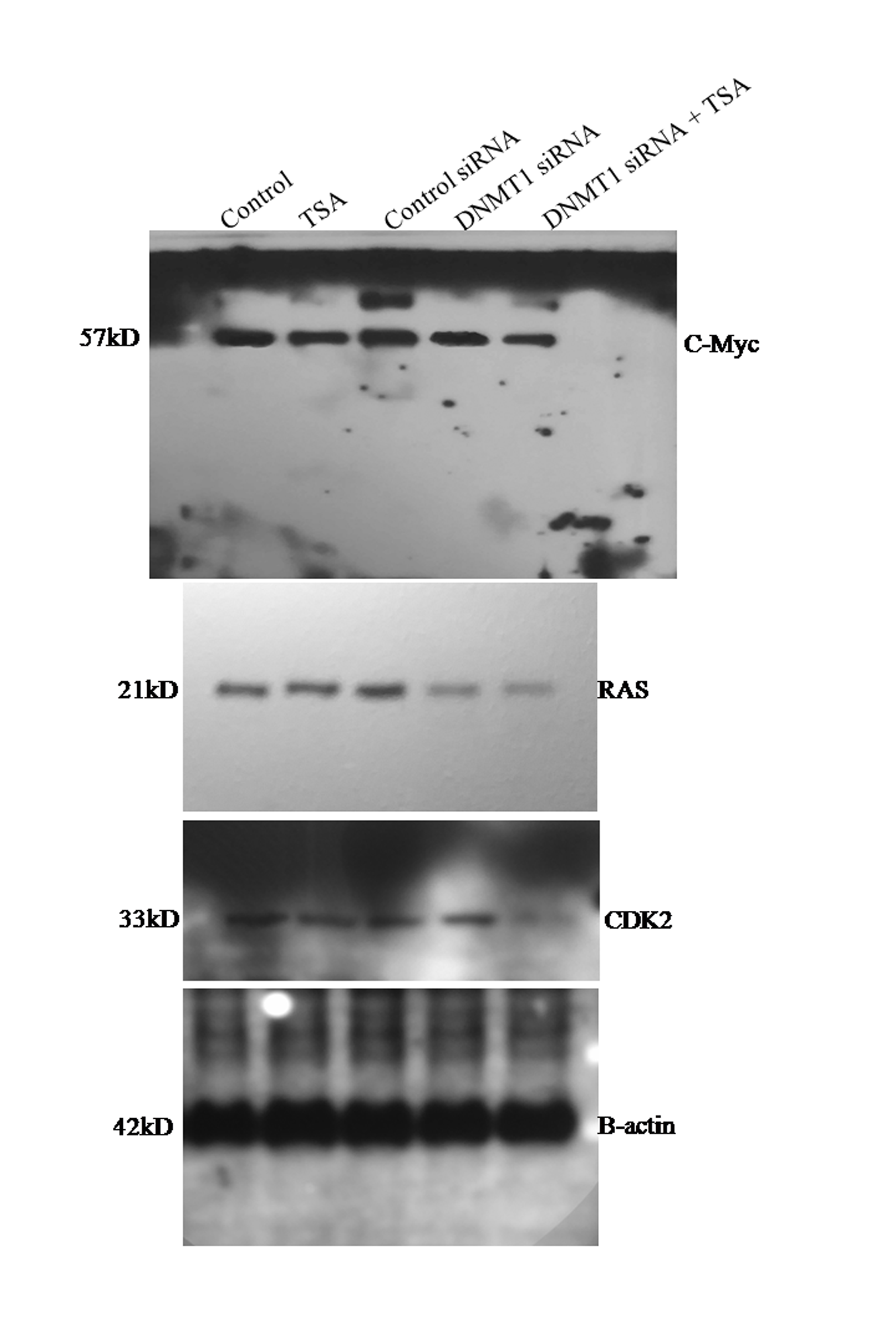

Supplement: Supplementary file 9 — Supplementary Information 9. [file 41598_2023_29712_MOESM9_ESM.tif]

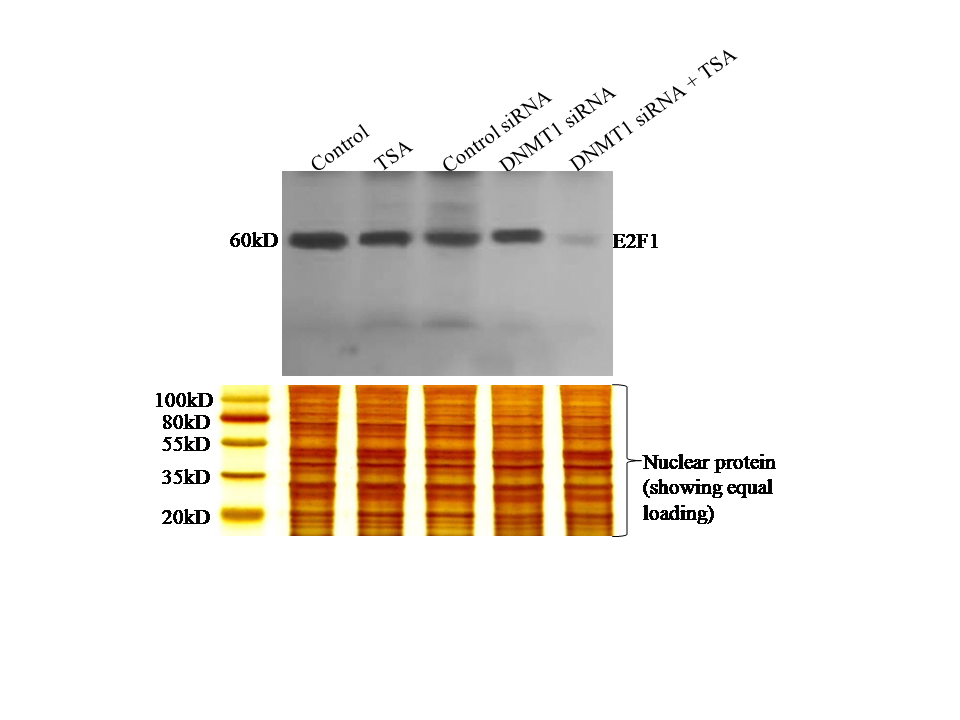

Supplement: Supplementary file 10 — Supplementary Information 10. [file 41598_2023_29712_MOESM10_ESM.tif]
