# Supplementary material for: Epigenetic alterations impede epithelial-mesenchymal transition by modulating centrosome amplification and Myc/RAS axis in triple negative breast cancer cells
Source: Sci Rep. 2023 Feb 11;13:2458. doi: 10.1038/s41598-023-29712-8 (PMC9922331; doi:10.1038/s41598-023-29712-8)
Supplement: Supplementary file 11 — Supplementary Information 11. [file 41598_2023_29712_MOESM11_ESM.docx]

**Supplementary Figure legends**

**Figure S2.**

The full-length original image of western blot results for Figure 1C. The chemiluminescent signals from the exposed membrane were recorded on X-ray films and the images of the X-Ray films (blots) were snapped and desired area of the blots were cropped and saved for further analysis. Anti-DNMT1 (sc20701) and anti-HDAC1 (sc8410) were obtained from Santa-Cruz Biotechnology anti-HDAC2 (ab137364) was obtained from abcam to analyze the expression of DNMT1, HDAC1 and HDAC2 respectively.

**Figure S3**

The full-length original image of western blot results for Figure 2B. The chemiluminescent signals from the exposed membrane were recorded on X-ray films and the images of the X-Ray films (blots) were snapped and desired area of the blots were cropped and saved for further analysis. Anti-γ Tubulin (ab16504) was obtained from abcam to analyze the expression of γ Tubulin.

**Figure S4**

The full-length original image of western blot results for Figure 3B. The chemiluminescent signals from the exposed membrane were recorded on X-ray films and the images of the X-Ray films (blots) were snapped and desired area of the blots were cropped and saved for further analysis. Anti-TUBGCP2 (ab140225) was obtained from abcam to analyze the expression of TUBGCP2.

**Figure S5**

The full-length original image of western blot results for Figure 4B. The chemiluminescent signals from the exposed membrane were recorded on X-ray films and the images of the X-Ray films (blots) were snapped and desired area of the blots were cropped and saved for further analysis. Anti-pericentrin (sc376111) was obtained from Santa-Cruz Biotechnology to analyze the expression of pericentrin.

**Figure S6**

The full-length original image of western blot results for Figure 5B. The chemiluminescent signals from the exposed membrane were recorded on X-ray films and the images of the X-Ray films (blots) were snapped and desired area of the blots were cropped and saved for further analysis. Anti-α Tubulin (ab52866) was obtained from abcam to analyze the expression of α Tubulin.

**Figure S7.**

The full-length original image of western blot results for Figure 8A. The chemiluminescent signals from the exposed membrane were recorded on X-ray films and the images of the X-Ray films (blots) were snapped and desired area of the blots were cropped and saved for further analysis. Anti-Bcl2 (ab196495), anti-Bax (ab289364) and anti-cleaved PARP (ab32064) were obtained from abcam to analyze the expression of Bcl2, Bax and cleaved PARP.

**Figure S8.**

The full-length original image of western blot results for Figure 9B. The chemiluminescent signals from the exposed membrane were recorded on X-ray films and the images of the X-Ray films (blots) were snapped and desired area of the blots were cropped and saved for further analysis. Anti-E cadherin (ab15148), anti-N cadherin (ab98952) and anti-vimentin (ab45939) were obtained from abcam to analyze the expression of E cadherin, N cadherin and vimentin.

**Figure S9.**

The full-length original image of western blot results for Figure 10A. The chemiluminescent signals from the exposed membrane were recorded on X-ray films and the images of the X-Ray films (blots) were snapped and desired area of the blots were cropped and saved for further analysis. Anti-c Myc (ab32072), anti-RAS (ab52939) and anti-CDK2 (ab32147) were obtained from abcam to analyze the expression of cMyc, RAS, CDK2.

**Figure S10.**

The full-length original image of western blot results for Figure 10C. The chemiluminescent signals from the exposed membrane were recorded on X-ray films and the images of the X-Ray films (blots) and silver stained gel (to show equal loading) were snapped and desired area of the blots were cropped and saved for further analysis. Anti-E2F1 (ab245308) was obtained from abcam to analyze the expression of E2F1.
